# Circular RNA circFBXW4 suppresses hepatic fibrosis via targeting the miR-18b-3p/FBXW7 axis

**DOI:** 10.7150/thno.42423

**Published:** 2020-03-26

**Authors:** Xin Chen, Hai-Di Li, Fang-Tian Bu, Xiao-Feng Li, Yu Chen, Sai Zhu, Jia-Nan Wang, Si-Yu Chen, Ying-Yin Sun, Xue-Yin Pan, Na-Na Yin, Jie-Jie Xu, Cheng Huang, Jun Li

**Affiliations:** 1 School of Pharmacy, Anhui Key Laboratory of Major Autoimmune Diseases, Anhui Institute of Innovative Drugs, Anhui Medical University, Hefei 230032, China.; 2 The Key Laboratory of Anti-inflammatory and Immune Medicines, Anhui Medical University, Ministry of Education, Hefei 230032, China.; 3 Institute for Liver Diseases of Anhui Medical University, ILD-AMU, Anhui Medical University, Hefei 230032, China.

**Keywords:** hepatic fibrosis, circular RNAs, circFBXW4, miR-18b-3p, FBXW7

## Abstract

**Rationale**: Circular RNAs (circRNAs) are a new form of noncoding RNAs that play crucial roles in various pathological processes. However, the expression profile and function of circRNAs in hepatic fibrosis (HF) remain largely unknown. In this study, we show a novel circFBXW4 mediates HF via targeting the miR-18b-3p/FBXW7 axis.

**Methods**: We investigated the expression profile of circRNAs, microRNAs and mRNAs in hepatic stellate cells (HSCs) from HF progression and regression mice by circRNAs-seq and microarray analysis. We found a significantly dysregulated circFBXW4 in HF. Loss-of-function and gain-of-function analysis of circFBXW4 were performed to assess the role of circFBXW4 in HF. Furthermore, we confirmed that circFBXW4 directly binds to miR-18b-3p by luciferase reporter assay, RNA pull down and fluorescence in situ hybridization analysis.

**Results**: We found that circFBXW4 downregulated in liver fibrogenesis. Enforcing the expression of circFBXW4 inhibited HSCs activation, proliferation and induced apoptosis, attenuated mouse liver fibrogenesis injury and showed anti-inflammation effect. Mechanistically, circFBXW4 directly targeted to miR-18b-3p to regulate the expression of FBXW7 in HF.

**Conclusions**: circFBXW4 may act as a suppressor of HSCs activation and HF through the circFBXW4/miR-18b-3p/FBXW7 axis. Our findings identify that circFBXW4 serves as a potential biomarker for HF therapy.

## Introduction

Chronic liver diseases are characterized by the pathology of liver fibrogenesis 1. Hepatic fibrosis (HF) results from the wound-healing response of liver exposed to persistent injury. Excessive accumulation of extracellular matrix (ECM) distorts the liver architecture by forming fibrous scars, and hepatocytes are replaced by abundant ECM. Hepatic stellate cells (HSCs) activate and transdifferentiate into myofibroblast-like cells, which are the major ECM-secreting cells and regulate ECM degradation in chronic liver fibrogenesis 2, 3. Additionally, the initiation of liver fibrogenesis is associated with inflammatory responses, with the stimulation of an array of fibrogenic actions in HSCs, including cell proliferation, migration, secretion of pro-inflammatory cytokine and synthesis of collagen 4, 5. Historically, HF was considered as a passive and irreversible pathology 6, while recent reports have demonstrated that HF is in fact a reversible pathology 7. Notably, spontaneous resolution of HF was achieved following the treatment of underlying disease in human, cessation of liver injury resulted in fibrosis regression in experimentally induced HF 8. Major mechanism of HF resolution is the removal of activated HSCs by apoptosis, increasing the activity of collagenolysis to degrade fibrillar collagen 9.

Noncoding RNAs are represented by circular RNAs (circRNAs), microRNAs (miRNAs) and long non-coding RNAs (lncRNAs). Recently, next-generation sequencing and bioinformatics technology reveal that circRNAs play crucial role in diagnose and prognosis of various diseases 10. The following characteristics of circRNAs have been reported: circRNAs are single-stranded transcripts generated by back-splicing 11, have covalently linked head-to-tail closed loop structures with neither 5'-3' polarity nor a polyadenylated tail 12, range in length from a few hundred to thousands of nucleotides, are widely expressed in mammals and show highly stability compared with linear RNAs 13, and exhibit a cell-type- or developmental-stage-specific expression pattern 14. Functions of circRNAs also have been identified, including act as miRNA sponges 15, binding to RNA-binding proteins 16 and protein decoys, being regulators of transcription 17. Interestingly, many circRNAs dysregulated in pathophysiological processes, circRNAs regulate expression of gene via acting as miRNA sponges, which are termed competitive endogenous RNA (ceRNA) mechanism 18. For example, circMTO1 harbors conventional miRNA binding sites and has been identified as an inhibitor of miRNA-9 in hepatocellular carcinoma (HCC) 19. In recent years, our group has focused on the regulation of ncRNAs on liver diseases. We found that miRNA-223 plays key role in liver injury and inflammation 20, miRNA-200a acts as a regulator in the initiation of HF 21. Furthermore, we reviewed advances in the involvement of lncRNAs in HF, with a specific focus on lncRNA-miRNA interaction 22, and the regulation of lncRNAs in HCC 23. Notwithstanding it has been reported that several circRNAs dysregulated in HF 24-26, expression profile, biological function, and molecular mechanism of circRNAs in HF, especially HSCs, remain largely unknown and need further investigation.

In this study, we analyzed the expression profile of circRNAs, miRNAs and mRNAs in HSCs from mice, attempting to identify biomarkers related to HF progression and regression pathological stages. We found a novel dysregulated circRNAs circFBXW4 which derived from the F box and WD 40 domain containing protein 4 (FBXW4) gene locus. Interestingly, circFBXW4 significantly downregulated in the liver fibrogenesis stage while rescued in the HF regression stage. Lower levels of circFBXW4 were also shown in HF patients compared with healthy controls. Functionally, overexpression of circFBXW4 inhibited the activation of HSCs, decreased myofibroblast transdifferentiation, attenuated mouse liver fibrogenesis injury, reduced collagen deposition and protected against inflammatory responses, suggesting the anti-fibrotic effects of circFBXW4 in HF. Mechanistically, we confirmed that circFBXW4 binds to miR-18b-3p, as a miRNA sponge, to regulate the expression of F box and WD 40 domain containing protein 7 (FBXW7), revealing that the circFBXW4/miR-18b-3p/FBXW7 axis plays crucial roles in HSCs activation and HF. Therefore, our study indicates that circFBXW4 may serve as a promising biomarker for HF therapy. To our knowledge, this is the first report investigates the expression profile, regulatory function and mechanism of circFBXW4 in HF.

## Results

### circRNAs expression profile in HSCs from mice by high-throughput sequencing

**Figure 1:**
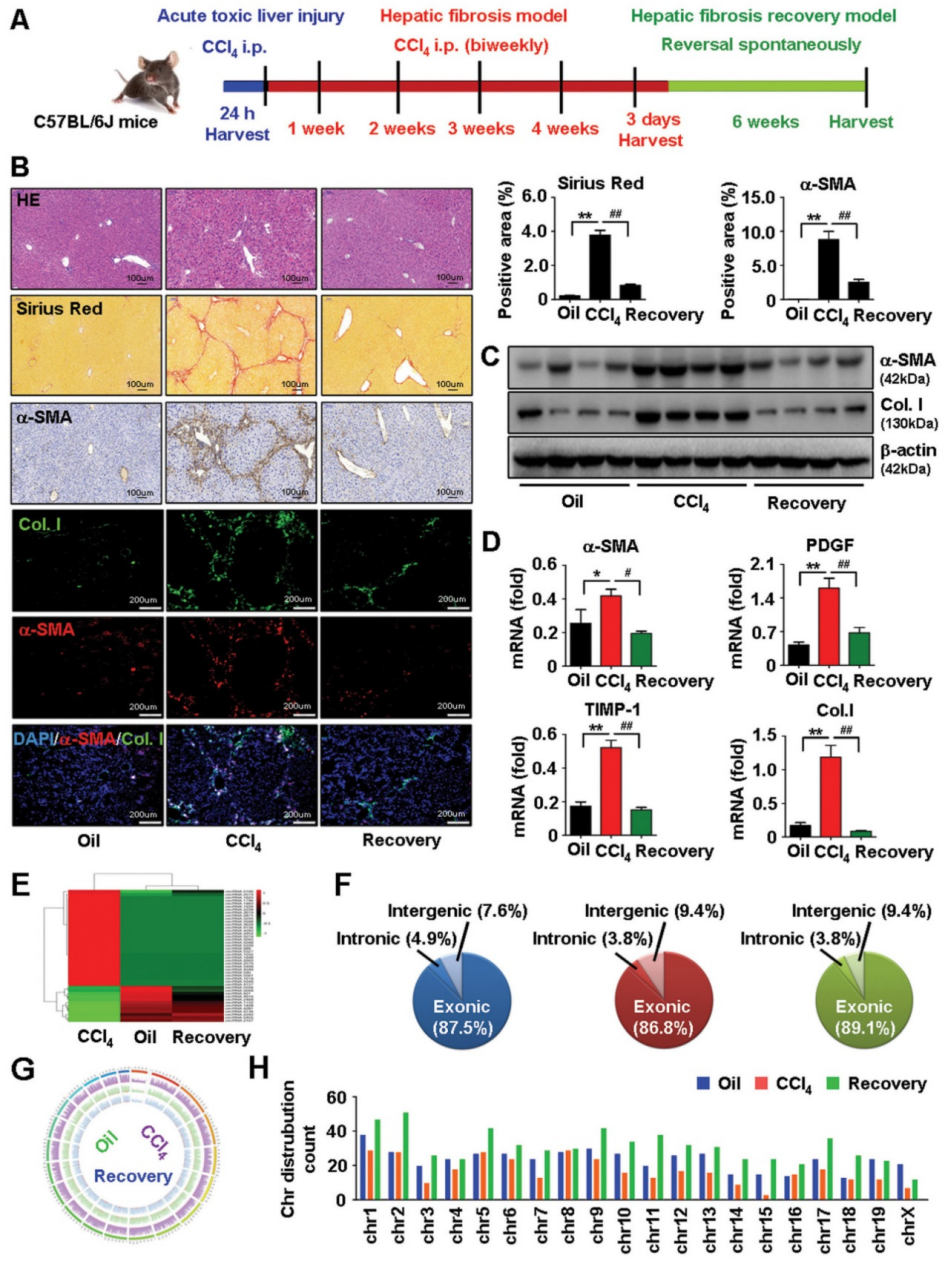
** circRNAs profiling in HSCs from vehicle, HF and HF recovery mice. (A)** The procedure of mouse acute toxic liver injury model, HF model and HF recovery model used for animal. **(B)** Pathology observation of H&E and sirius red staining of liver tissues, scale bar, 100 μM; immunohistochemistry staining for α-SMA in liver tissues, scale bar, 100 μM; immunofluorescence staining of α-SMA and collagens I in liver tissues, nuclei were stained with DAPI, scale bar, 200 μM. The positive staining areas were measured by Ipwin32 software. **(C)** Protein expression of α-SMA and collagens I in HSCs detected by western blot. **(D)** mRNA levels of α-SMA, PDGF, TIMP-1 and collagens I in HSCs detected by qRT-PCR. **(E)** A heatmap showed differentially expressed circRNAs with same expression pattern in vehicle and HF recovery mice, which opposite to HF mice. **(F)** circRNA composition in terms of genes distribution. **(G, H)** The coverage and distribution of differentially expressed circRNAs on the mouse chromosomes. Data represent the mean ± s.e.m (n=8). *p<0.05, **p<0.01 versus Oil; ^#^*p*<0.05, ^##^*p*<0.01 versus CCl_4_.

Activation of HSCs is a critical event in the development and maintenance of HF 1, 27. To investigate the expression profile of circRNAs involved in HF, primary HSCs (isolated from vehicle, HF progression and HF regression mice) were analyzed by circular RNA high-throughput sequencing (Seq). The procedure used for animal treatment is shown in Figure 1A. First, we successfully established the mouse model of carbon tetrachloride (CCl_4_)-induced HF and HF spontaneous regression. Results showed that the pathological characteristics of liver fibrogenesis and injury were aggravated in HF mice while regressed in HF recovery mice (Figure 1B and Figure S1A). Additionally, the expression of fibrogenic factors consistently increased in HSCs from HF mice while reduced in HF recovery mice (Figure 1C-D). Furthermore, we detected a total of 4343 differentially expressed circRNAs, among them, 539 differentially expressed circRNAs were reported in the circBase. Results showed that 154 circRNAs were differentially expressed in HF compared with vehicle mice, and 77 circRNAs were differentially expressed in HF recovery compared with HF mice (File S1-2). Notably, we found 12 circRNAs downregulated in HF mice while their expression rescued in HF recovery mice, and 32 circRNAs upregulated in HF while suppressed in HF recovery mice (File S3). circRNAs with the same expression pattern in vehicle and HF recovery mice, which was opposite to the HF mice may potentially relate to HF progression and regression. Heatmaps reflected the differentially expressed circRNAs was showed in Figure 1E. In addition, the composition of differentially expressed circRNAs in terms of gene distribution was showed in Figure 1F. The coverage and distribution of differentially expressed circRNAs on the mouse chromosomes were presented in Figure 1G-H.

### Expression of circFBXW4 decreased in HF while rescued in HF recovery process

**Figure 2:**
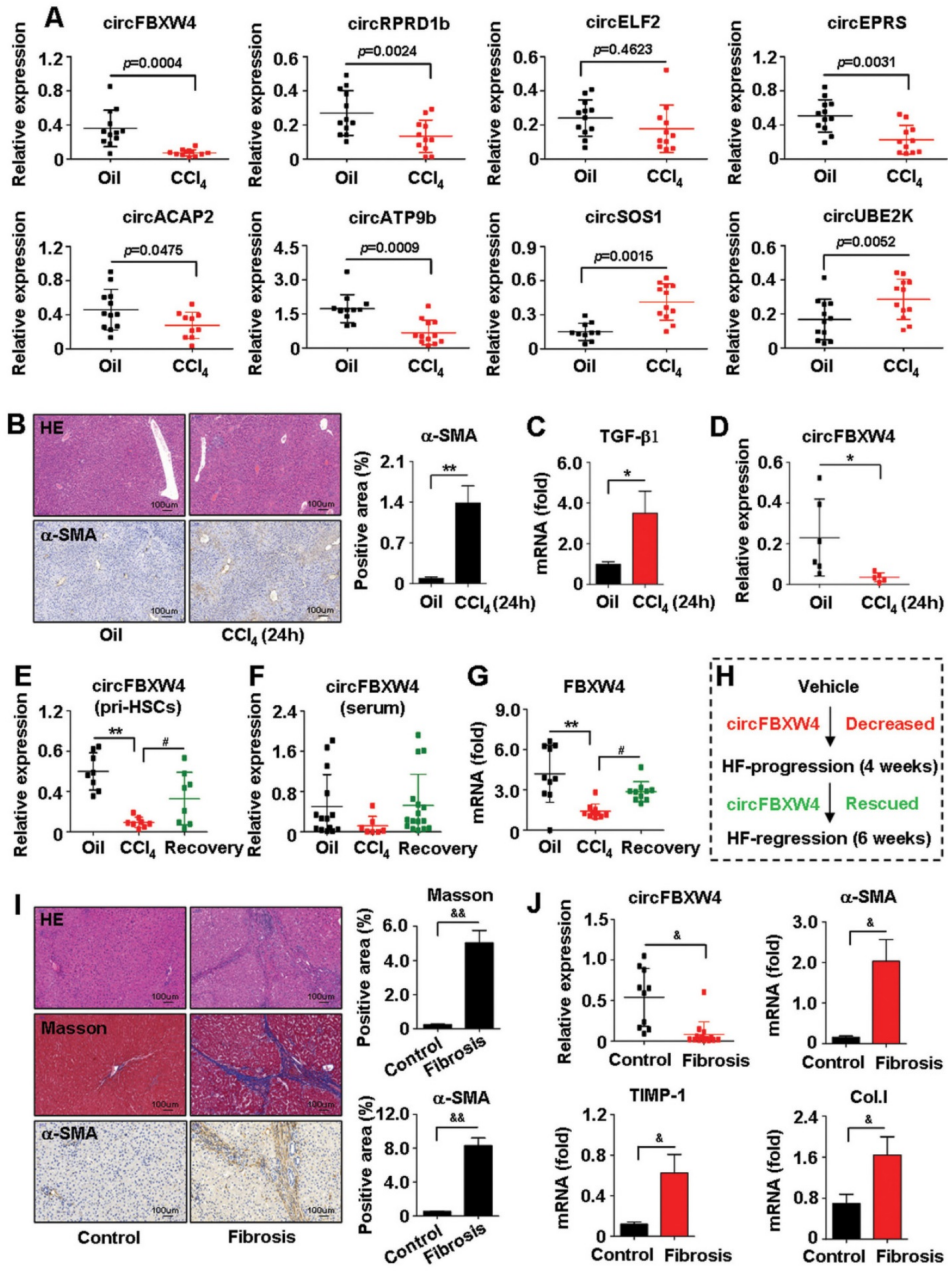
** Expression of circFBXW4 decreased in HF and rescued in HF recovery mice. (A)** Eight differentially expressed circRNAs confirmed by qRT-PCR in HSCs (n=10-12). **(B)** Pathology observation of H&E staining from acute toxic liver injury tissue, scale bar, 100 μM; immunohistochemistry staining for α-SMA, scale bar, 100 μM. The positive staining areas were measured by Ipwin32 software.** (C)** mRNA level of TGF-β1 and** (D)** relative expression of circFBXW4 in HSCs (n=6). **(E)** Relative expression of circFBXW4 in HSCs and **(F)** in serum. **(G)** mRNA level of FBXW4 in HSCs. **(H)** Expression pattern of circFBXW4 in HF progression and regression stages. **(I)** Pathology observation of H&E and masson staining of human liver tissue, scale bar, 100 μM; immunohistochemistry staining for α-SMA, scale bar, 100 μM. The positive staining areas were measured by Ipwin32 software.** (J)** Relative expression of circFBXW4, α-SMA, TIMP-1 and collagens I in human liver tissue detected by qRT-PCR (Control, n=10; Fibrosis, n=14). Data represent the mean ± s.e.m. *p<0.05, **p<0.01 versus Oil; ^#^*p*<0.05, ^##^*p*<0.01 versus CCl_4_; ^&^*p*<0.05, ^&&^*p*<0.01 versus Control.

Majority of circRNAs are derived from the exonic region of known protein-coding genes by back-splicing 28. Through sorting based on circRNAs expression intensity and screening in terms of the exon type, we selected 27 differentially expressed circRNAs, including 11 circRNAs upregulated and 16 circRNAs downregulated in liver fibrogenesis (Table S1). Results of qRT-PCR confirmed the top 8 dysregulated circRNAs, which was consistent with the circRNA-seq data. We focused on circFBXW4, which significantly downregulated in HF compared with vehicle (Figure 2A and Figure S1B). Additionally, we also established a model of mouse acute liver injury (Figure 2B-C) and we found that circFBXW4 decreased in mice exposed to acute liver injury (Figure 2D). Next, we confirmed that expression of circFBXW4 decreased in liver fibrogenesis while rescued in HF recovery process (Figure 2E). Report suggests that the levels of circRNAs in peripheral blood could be used as diagnostic biomarkers for diseases 29. Thus, we further detected the levels of circFBXW4 in serum and found a similar expression pattern of circFBXW4 in HSCs (Figure 2F). Consistently, FBXW4, the host gene of circFBXW4, decreased in HF and restored in HF recovery process (Figure 2G). Additionally, we employed another well-established bile duct ligation (BDL) HF model (Figure S1C). circFBXW4 attenuated in BDL-operated mice for 15 days compared with Sham mice (Figure S1D), this is consistent to the result that circFBXW4 decreased following the elevation of fibrotic factors and liver injury (Figure S1E-F). Furthermore, we found that levels of circFBXW4 decreased in human fibrotic liver tissues compared with healthy controls (Figure 2I-J). Together, these results reveal the downregulation of circFBXW4 in HF progression while restoration of circFBXW4 in HF regression (Figure 2H). Indicating that expression of circFBXW4 associates with HF pathology and potential value of circFBXW4 serves as a diagnostic and prognostic marker for HF.

### Characterizations of circFBXW4

**Figure 3:**
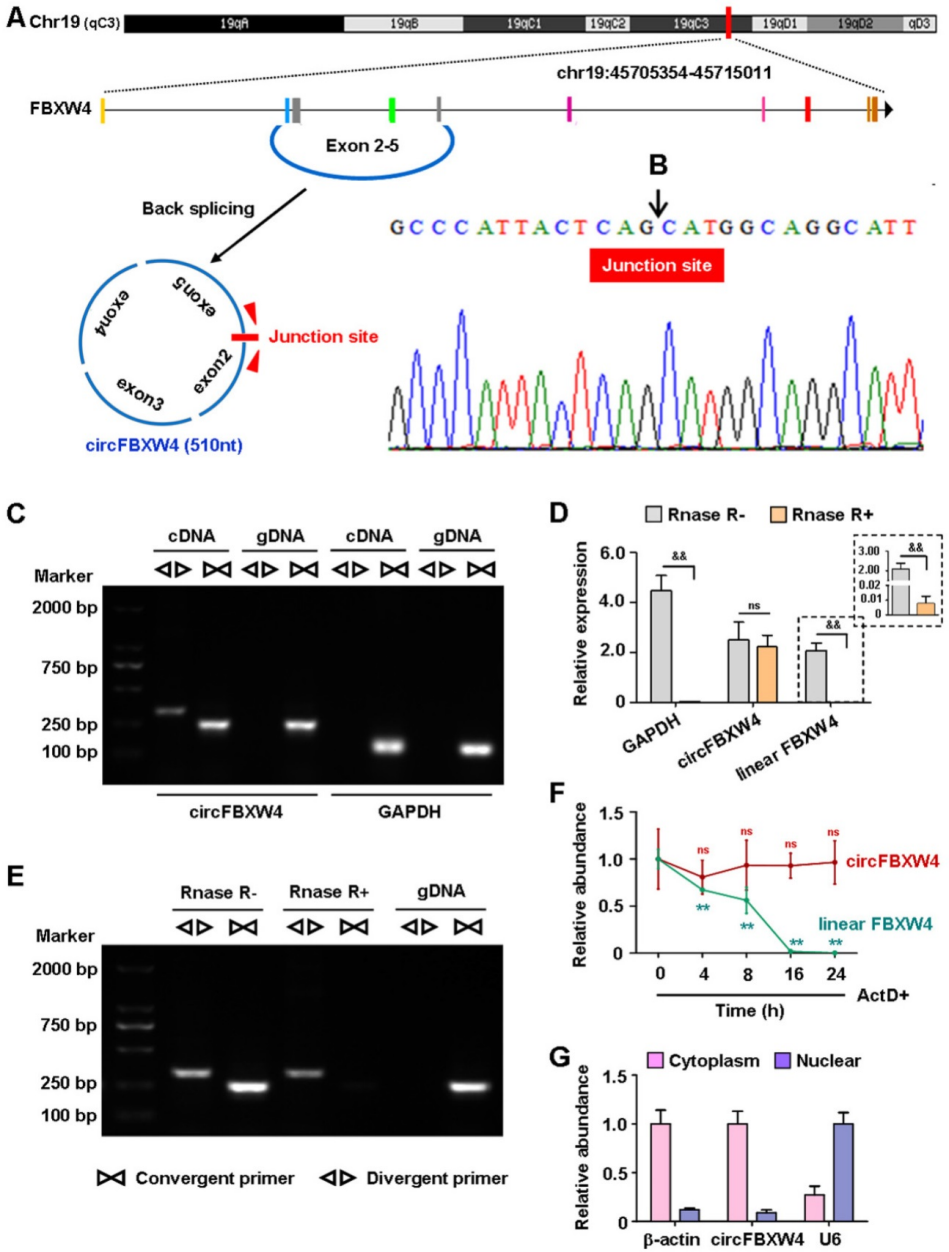
** Characterizations of circFBXW4. (A)** Schematic diagram showed the genomic location and back-splicing pattern of circFBXW4. **(B)** Sanger sequencing of circFBXW4, the arrow represents the “head-to-tail” splicing site. **(C)** Divergent primers amplified circFBXW4 from cDNA by PCR and an agarose gel electrophoresis, rather than from gDNA, GAPDH was used as a linear control (n=3). **(D)** circFBXW4, rather than linear FBXW4 or GAPDH, resisted to Rnase R digestion (n=6).** (E)** circFBXW4 from cDNA was detected with divergent primers even exposed to Rnase R digestion, the opposite result showed from gDNA (n=3). **(F)** Relative expression of circFBXW4 and linear FBXW4 in HSCs treated with ActD+ (actinomycin D) at the indicated time points (n=6). **(G)** Cytoplasmic and nuclear fractionation assay revealed that circFBXW4 mainly localized in the cytoplasm (n=4). Data represent the mean ± s.e.m. ^&^*p*<0.05, ^&&^*p*<0.01 versus Rnase R- group; *p<0.05, **p<0.01 versus time 0.

circFBXW4 (mm9_circ_000338) derives from the host gene FBXW4, termed circFBXW4 in this study. circFBXW4 locates on chromosome 19: 45705354- 45715011 (510 nt), the genomic structure suggests that circFBXW4 consists of 4 exons (exon 2-5) from the FBXW4 gene locus (Figure 3A). The RT-PCR product of circFBXW4 was confirmed to involve head-to-tail splicing and matched the sequence reported for circFBXW4 in circBase, as obtained using Sanger sequencing (Figure 3B). In addition, divergent and convergent primers were designed for circFBXW4, complementary DNA (cDNA) and genomic DNA (gDNA) were used as templates, which were confirmed by 1% agarose gel electrophoresis. Results showed that a single and distinct product of expected size was amplified using circFBXW4 divergent primers (a 318 bp fragment) from only cDNA, while there was no amplification product from gDNA (Figure 3C). As a head-to-tail splicing product may also derive from genomic rearrangements or trans-splicing, to further confirm the characteristics of circFBXW4, we examined the resistance of circFBXW4 to Rnase R digestion (Figure 3E), a highly processive 3'-5' exonuclease 30. Results confirmed that circFBXW4 exhibited abundant and stable features in HSCs (Figure 3D). Actinomycin D, an inhibitor of transcription, was added to HSCs at the indicated times, and the transcript half-life of circFBXW4 exceeded 24 h, while that of linear FBXW4 was approximately 10 h (Figure 3F). Suggesting that circFBXW4 was more stable than the corresponding linear transcript. In addition, circFBXW4 predominantly localized in the cytoplasm evaluated by cytoplasmic and nuclear fractionation assay, β-actin and U6 were applied as positive controls in the cytoplasm and nucleus, respectively (Figure 3G).

### circFBXW4 suppresses activation and proliferation of LX-2 cells *in vitro*

To assess the functional roles of circFBXW4 in LX-2 cells (a human HSC line with the key features of activated HSCs) 31, loss-of-function and gain-of- function assays were performed, respectively. First, we determined the circFBXW4 (hsa_circ_0008362) sequences (Figure S2A), circular RNA characteristics and stability of circFBXW4 in LX-2 cells (Figure S2B and S2D). Results of fluorescence in situ hybridization (FISH) showed that circFBXW4 mainly resided in the cytoplasm (Figure S2C). Next, we constructed two siRNAs target to the back-splicing site of circFBXW4 (Figure S2E) and confirmed the silencing efficiency of si-circFBXW4 in LX-2 cells after transfection (Figure S2F). Results showed that knockdown of circFBXW4 exhibited negligible effect on the FBXW4 linear species (Figure S2F). Functionally, knockdown of circFBXW4 subsequently elevated the mRNA levels of fibrogenic factors transforming growth factor-β1 (TGF-β1) and alpha-smooth muscle actin (α-SMA), and increased the protein expression of α-SMA and collagen I in LX-2 cells (Figure S2F-G). Moreover, knockdown of circFBXW4 promoted cell proliferation (Figure S2H) and reduced the proportion of cells in G_0_/G_1_ phase (Figure S2I), suggesting that decrease of circFBXW4 enhanced the cell cycle in LX-2 cells.

**Figure 4:**
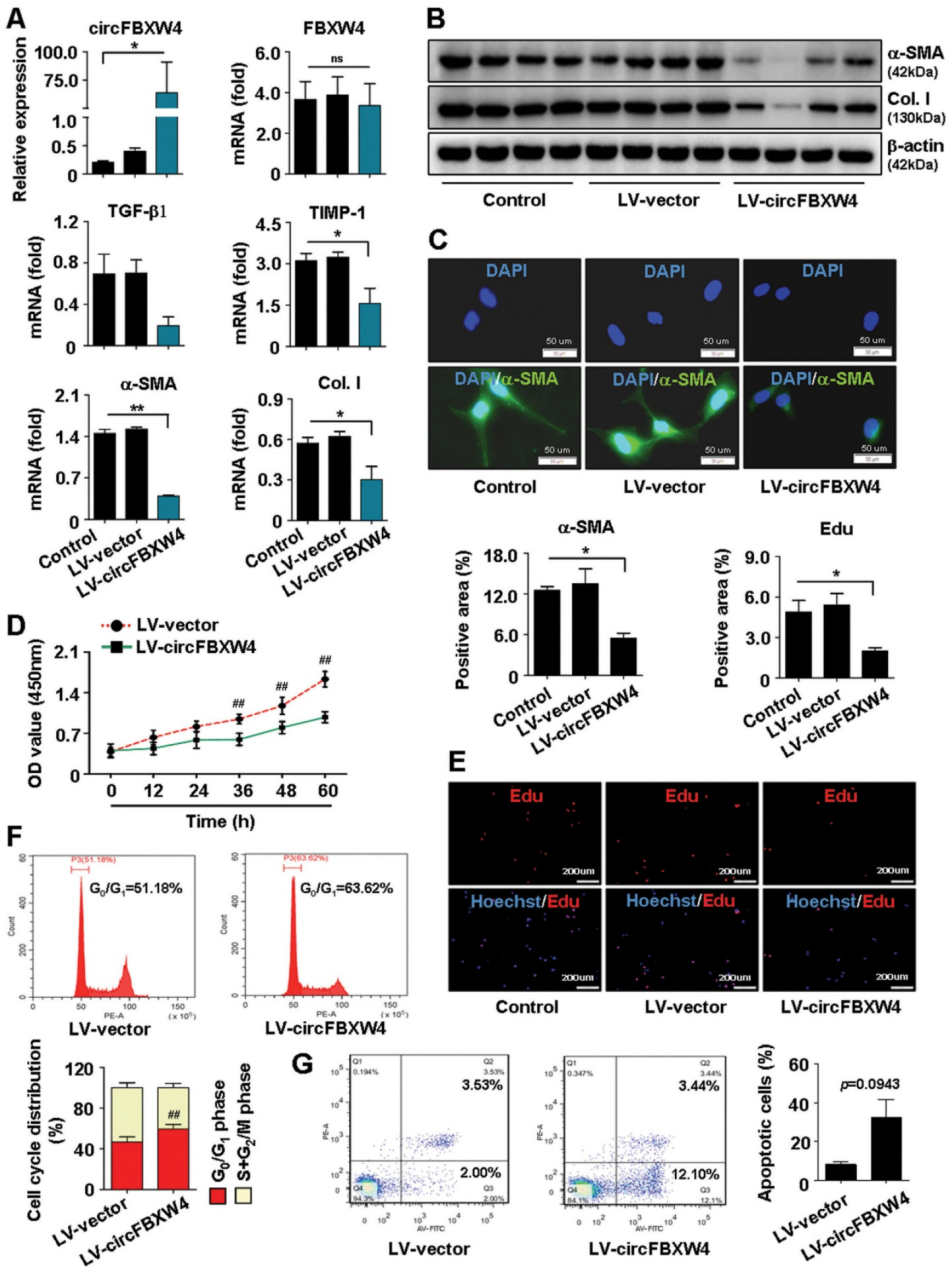
** Effects of circFBXW4 overexpression on the activation, proliferation and apoptosis of LX-2 cells. (A)** Stable overexpression efficiency of circFBXW4, and mRNA levels of FBXW4, TGF-β1, TIMP-1, α-SMA and collagens I detected by qRT-PCR. **(B)** Protein expression of α-SMA and collagens I in LX-2 cells (n=4).** (C)** Immunofluorescence staining for α-SMA, nuclei were stained with DAPI, scale bar, 50 μM.** (D)** Effect of circFBXW4 overexpression on the proliferation of LX-2 cells detected by CCK8 assay at the indicated time points (n=6).** (E)** Assessment of DNA synthesis using Edu assay in LX-2 cells, nuclei were stained with Hoechst33342, scale bar, 200μM.** (F)** Flow cytometry showed the cell cycle distribution in G_0_/G_1_ phase increased following overexpression of circFBXW4.** (G)** Overexpression of circFBXW4 induced apoptosis of LX-2 cells with indicated treatment. Data represent the mean ± s.e.m. *p<0.05, **p<0.01 versus Control; ^#^*p*<0.05, ^##^*p*<0.01 versus LV-vector.

In contrast, stable overexpression of circFBXW4 exerted the opposite effects on LX-2 cells. Stable circFBXW4 overexpressing cells were established by Lentivirus-circFBXW4 (LV-circFBXW4) transfected into LX-2 cells followed by puromycin (1 μg/ml) screening for 1 week. Overexpression efficiency of circFBXW4 was determined and the level of circFBXW4 was approximately increased 300-fold in LV-circFBXW4 treated LX-2 cells compared with control (Figure 4A). Functionally, overexpression of circFBXW4 reduced the mRNA levels of fibrogenic factors TGF-β1, tissue inhibitor of metallopeptidase-1 (TIMP-1), α-SMA and collagen I, decreased the protein expression of α-SMA and collagen I (Figure 4A-B). Enhancing circFBXW4 levels suppressed the activation of LX-2 cells (Figure 4C). Consistently, overexpression of circFBXW4 decreased cell proliferation and DNA synthesis (Figure 4D-E), induced an arrest of the cell cycle in LX-2 cells (Figure 4F). In addition, enhancing circFBXW4 levels induced apoptosis of LX-2 cells (Figure 4G).

### Anti-fibrotic effects of circFBXW4 in HF mice* in vivo*

**Figure 5:**
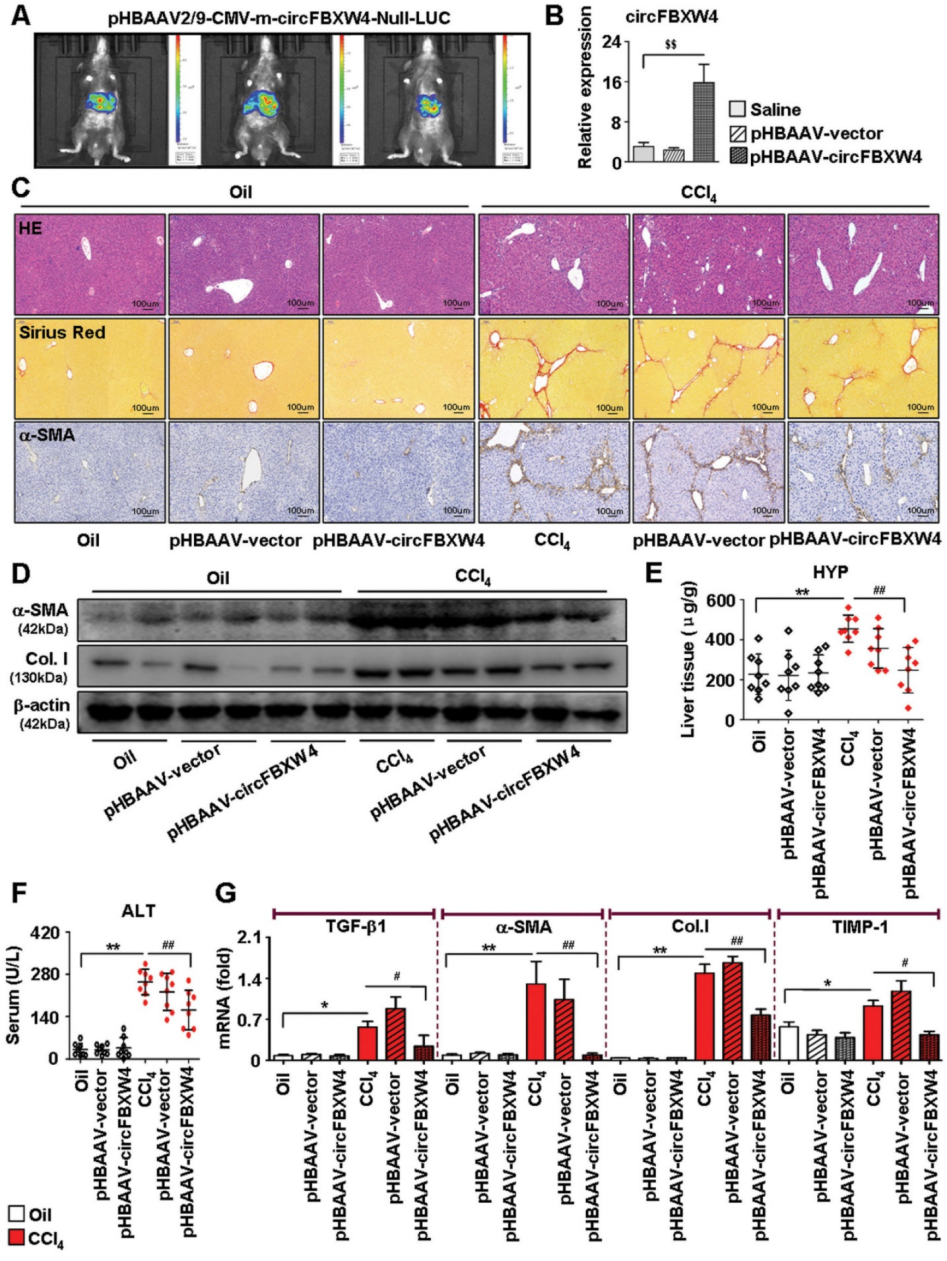
** Anti-fibrotic effects of circFBXW4 in HF mice. (A)** Mouse *in vivo* imaging analysis showed luciferase-labelled pHBAAV-circFBXW4 was specific located in mouse liver tissue.** (B)** Overexpression efficiency of circFBXW4 in HSCs from mice following pHBAAV-circFBXW4 administration. **(C)** Pathology observation of H&E staining, sirius red staining and immunohistochemistry staining for α-SMA in liver tissue, scale bar, 100 μM. The positive staining areas were measured by Ipwin32 software.** (D)** Protein expression of α-SMA and collagens I in HSCs from mice after circFBXW4 overexpression.** (E, F)** Test of HYP in mouse liver tissue and ALT in serum. **(G)** mRNA levels of TGF-β1, α-SMA, collagens I and TIMP-1 in HSCs from mice following pHBAAV-circFBXW4 treatment. Data represent the mean ± s.e.m (n=8). ^$^*p*<0.05, ^$$^*p*<0.01 versus Saline; *p<0.05, **p<0.01 versus Oil; ^#^*p*<0.05, ^##^*p*<0.01 versus CCl_4_.

Next, we further investigated the effects of circFBXW4 on HF mice. We injected luciferase-labelled pHBAAV-circFBXW4 into the tail vein of mice and determined the liver-tissue-specific location of pHBAAV-circFBXW4 by *in vivo* imaging analysis (Figure 5A). We detected the overexpression efficiency of circFBXW4 in three different cell types in the liver (Figure 5B and Figure S3A-D). Functionally, liver parenchyma and vascular architecture distortion, collagen deposition and α-SMA^+^ myofibroblasts immune signal were consistently reduced in HF mice following pHBAAV-circFBXW4 administration (Figure 5C), a corresponding with the decreased protein expression of α-SMA and collagen I in HSCs (Figure 5D). Both level of hydroxyproline (HYP) in liver tissue and alanine aminotransferase (ALT) in serum were reduced in pHBAAV-circFBXW4-treated HF mice (Figure 5E-F). Moreover, fibrogenic factors (TGF-β1, α-SMA, collagen I and TIMP-1) were downregulated following circFBXW4 overexpression (Figure 5G). As shown in Figure S3E, we further evaluated the effects of circFBXW4 on proliferation and apoptosis of HSCs, which was indicated by decreasing c-Myc and CyclinD1 (cell-cycle-related factors) protein expression and increasing the ratio of Bax/Bcl-2 (apoptosis-related factors). Taken together, these results demonstrated that liver fibrogenesis injury and expression of fibrogenic markers were significantly suppressed in HF mice with circFBXW4 overexpression by delivery of liver-specific pHBAAV-circFBXW4.

### circFBXW4 protects against inflammatory responses in HF mice* in vivo*

**Figure 6:**
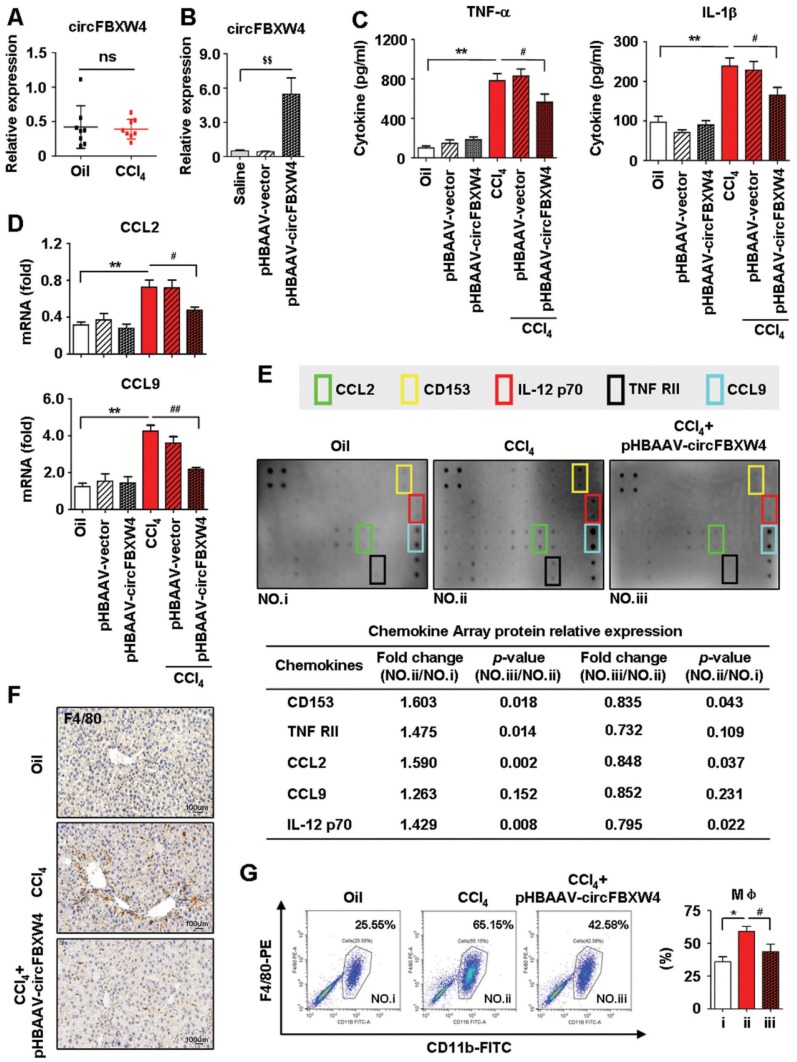
** Anti-inflammation of circFBXW4 in HF mice. (A)** Expression pattern of circFBXW4 in liver macrophages from vehicle and HF mice. **(B)** pHBAAV-circFBXW4 successful elevated circFBXW4 level in liver macrophages. **(C)** Levels of pro-inflammatory cytokines TNF-α and IL-1β in serum detected by ELISA. **(D)** mRNA levels of CCL2 and CCL9 in liver macrophages (n=8). **(E)** The up panel is a schematic representation of mouse inflammation antibody array containing 40 inflammation antibodies, positive control, negative control and blank spots with duplicates. Array showed the expression of inflammatory factors in mouse liver lysates, fold changes of differentially expressed 5 inflammatory factors (CCL2, CD153, IL-12 p70, TNF-RII, CCL9) were showed in the down panel. **(F)** Immunohistochemistry staining for F4/80 in liver tissue, scale bar, 50 μM. The positive staining areas were measured by Ipwin32 software.** (G)** Flow cytometric analysis showed the reduction of F4/80^+^CD11b^+^ liver macrophages in HF mice with pHBAAV-circFBXW4 treatment (n=4). Data represent the mean ± s.e.m. ^$^*p*<0.05, ^$$^*p*<0.01 versus Saline; *p<0.05, **p<0.01 versus Oil; ^#^*p*<0.05, ^##^*p*<0.01 versus CCl_4_.

Inflammatory responses exhibit important effects on the initiation of HSCs activation and HF, therefore, the functions of circFBXW4 on inflammatory responses in liver fibrogenesis was assessed. We found that levels of circFBXW4 exhibited negligible change in liver macrophages from HF mice compared with vehicle (Figure 6A), and circFBXW4 levels was significantly elevated in liver macrophages after pHBAAV-circFBXW4 administration (Figure 6B). Interestingly, the circulating levels of pro-inflammatory cytokines TNF-α and IL-1β were reduced following circFBXW4 overexpression (Figure 6C). Furthermore, mRNA levels of C-C motif chemokine ligand 2 (CCL2) and CCL9 were subsequently suppressed in liver macrophages treated with pHBAAV-circFBXW4 (Figure 6D). We further tested the expression of 40 inflammatory factors in mouse liver tissue lysates using an inflammatory antibody array. Results showed that the levels of five inflammatory factors (CCL2, CD153, IL-12 p70, TNF RII, CCL9) were increased in HF mice compared with vehicle, while they were decreased following circFBXW4 overexpression (Figure 6E). Additionally, immunostaining of F4/80 was consistently reduced in liver tissue after delivery of pHBAAV-circFBXW4 delivery (Figure 6F). F4/80^+^CD11b^+^ liver macrophages (hepatic macrophages and infiltrating monocytes) were consistently decreased in HF mice following pHBAAV-circFBXW4 treatment (Figure 6G). Parallelly, we characterized pHBAAV-circFBXW4 treated HF mice showed a higher proportion of M2 phenotype macrophages (F4/80^+^/CD206^+^) (Figure S4A). Additionally, both mRNA level of IL-10 in liver macrophages and circulating levels of IL-10 in serum were increased in pHBAAV-circFBXW4 treated HF mice (Figure S4B-C). Our previous study confirmed that IL-10 suppresses HSCs activation, and IL-10 is known to regulate inflammatory responses and to direct a sustained wound-healing response in HF 1. Consistently, level of IL-10R was elevated in HSCs after circFBXW4 overexpression (Figure S4D), and the expression of α-SMA, TGF-β1 and TIMP-1 was inhibited in HSCs exposed to IL-10 (Figure S4E). These results indicate that circFBXW4 triggered the anti-inflammatory cytokine IL-10 to mediate the activation of HSCs. Suggesting that circFBXW4 promoted the M2 macrophage phenotype, which prevented the exacerbation of inflammation and substantial fibrogenesis damage in HF. Moreover, liver crucially participates in systemic inflammatory responses, thus, we further detected the level of circFBXW4 in different organs (heart, spleen, lung and kidney) (Figure S5A). Interestingly, liver-specific administration of pHBAAV-circFBXW4 showed anti-inflammation effects in heart, spleen, lung and kidney by mediating pro-inflammatory and/or anti-inflammatory cytokines in response to immune signals (Figure S5B-E).

### Microarray analysis and identification of circFBXW4-miR-18b-3p connectivity

**Figure 7:**
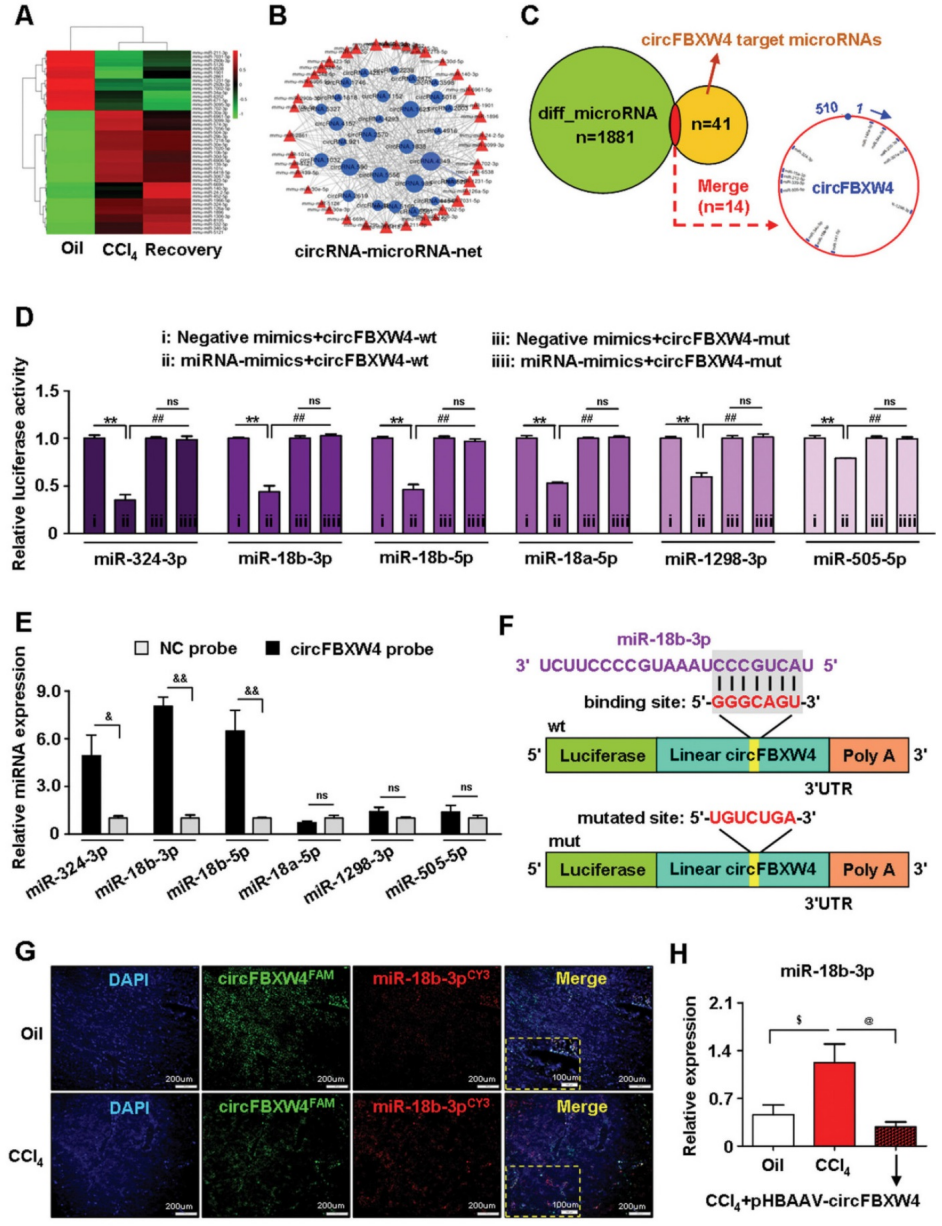
** Microarray analysis of HSCs and circFBXW4 binds miR-18b-3p. (A)** Heatmap of differentially expressed miRNAs in HSCs from vehicle, HF and HF recovery mice. **(B)** circRNA-miRNA network showed differentially expressed miRNAs with their circRNA targets. **(C)** Differentially expressed miRNAs combine with circFBXW4 predicted miRNA targets, and a schematic showed the binding sites of miRNAs bind to circFBXW4.** (D)** The renilla luciferase activity analysis of wild type or mutant circFBXW4 and miRNAs mimics or negative control co-transfected into HEK-293T cells, respectively. Data are presented as the ratio of renilla luciferase activity to firefly activity (n=3).** (E)** Biotinylated circFBXW4 probe could capture 3 miRNA candidates by RNA pull-down analysis (n=4).** (F)** Schematic of miR-18b-3p sites in circFBXW4 based on complementary sequences.** (G)** Co-localization of circFBXW4 with miR-18b-3p was determined in mouse liver tissue by FISH, nuclei were stained with DAPI, scale bar, 200 μM and 100 μM.** (H)** Level of miR-18b-3p was suppressed in HSCs following circFBXW4 overexpression (n=7). Data represent the mean ± s.e.m. *p<0.05, **p<0.01 versus Negative mimics+circFBXW4-wt; ^#^*p*<0.05, ^##^*p*<0.01 versus miRNA-mimics+circFBXW4-wt; ^&^*p*<0.05, ^&&^*p*<0.01 versus circFBXW4 probe; ^$^*p*<0.05, ^$$^*p*<0.01 versus Oil; ^@^*p*<0.05, ^@@^*p*<0.01 versus CCl_4_.

A feature of circRNAs is acts as miRNAs sponges. To assess the potential miRNAs bind to circFBXW4, and identify promising novel miRNAs relate to HF, miRNA expression profile in HSCs was analyzed by microarray. We detected a total of 1881 differentially expressed miRNAs, among them, 132 miRNAs differentially expressed in HF mice compared with vehicle mice, and 160 miRNAs differentially expressed in HF recovery mice compared with HF mice (File S4-5). Importantly, we found 26 miRNAs downregulated in HF mice while rescued in HF recovery mice, along with 15 miRNAs upregulated in HF mice while suppressed in HF recovery mice (File S6). The dysregulated miRNAs were presented in a heatmap showed in Figure 7A. Network based on the correlations between differentially expressed miRNAs and their differentially expressed circRNA targets was showed in a diagram (Figure 7B). Based on sequence pairing, circFBXW4 potentially targeted 41 miRNAs using miRanda program. We analyzed 14 miRNA candidates showed in the schematic (Figure 7C). Next, a circFBXW4 fragment with wild-type or mutant complementary binding sites was inserted into the luciferase reporter gene psiCHECK-2, with miRNA mimics and a negative control were also constructed. Results showed that the renilla luciferase activity of circFBXW4-wt was significantly inhibited in 6 miRNA (miR-324-3p, miR-18b-3p, miR-18b-5p, miR-18a-5p, miR-1298-3p and miR-505-5p) mimics groups compared with negative control (Figure 7D). Subsequently, we purified the circFBXW4-bound RNA complexes and confirmed the enrichment of 3 miRNAs candidates, RNA pull down assay suggested that miR-18b-3p, miR-324-3p and miR-18b-5p may directly bind to circFBXW4 (Figure 7E). Binding sites of miR-18b-3p were identified within the circFBXW4 sequences (Figure 7F). Additionally, we found circFBXW4 and miR-18b-3p predominantly co-localized in the cytoplasm detected by FISH assay (Figure 7G). Moreover, microarray analysis revealed that levels of miR-18b-3p significantly increased in HF mice compared with vehicle, which was confirmed by qRT-PCR (Figure 7H). Interestingly, higher level of miR-18b-3p in liver fibrogenesis was suppressed following pHBAAV-circFBXW4 administration. Together, circFBXW4 directly acted as a sponge of miR-18b-3p, and circFBXW4 regulated the levels of miR-18b-3p in HF (Figure 7H). Therefore, a focus was placed on the interaction between circFBXW4 and miR-18b-3p for further investigation.

### circFBXW4 upregulates the expression of FBXW7 by sponging miR-18b-3p

**Figure 8:**
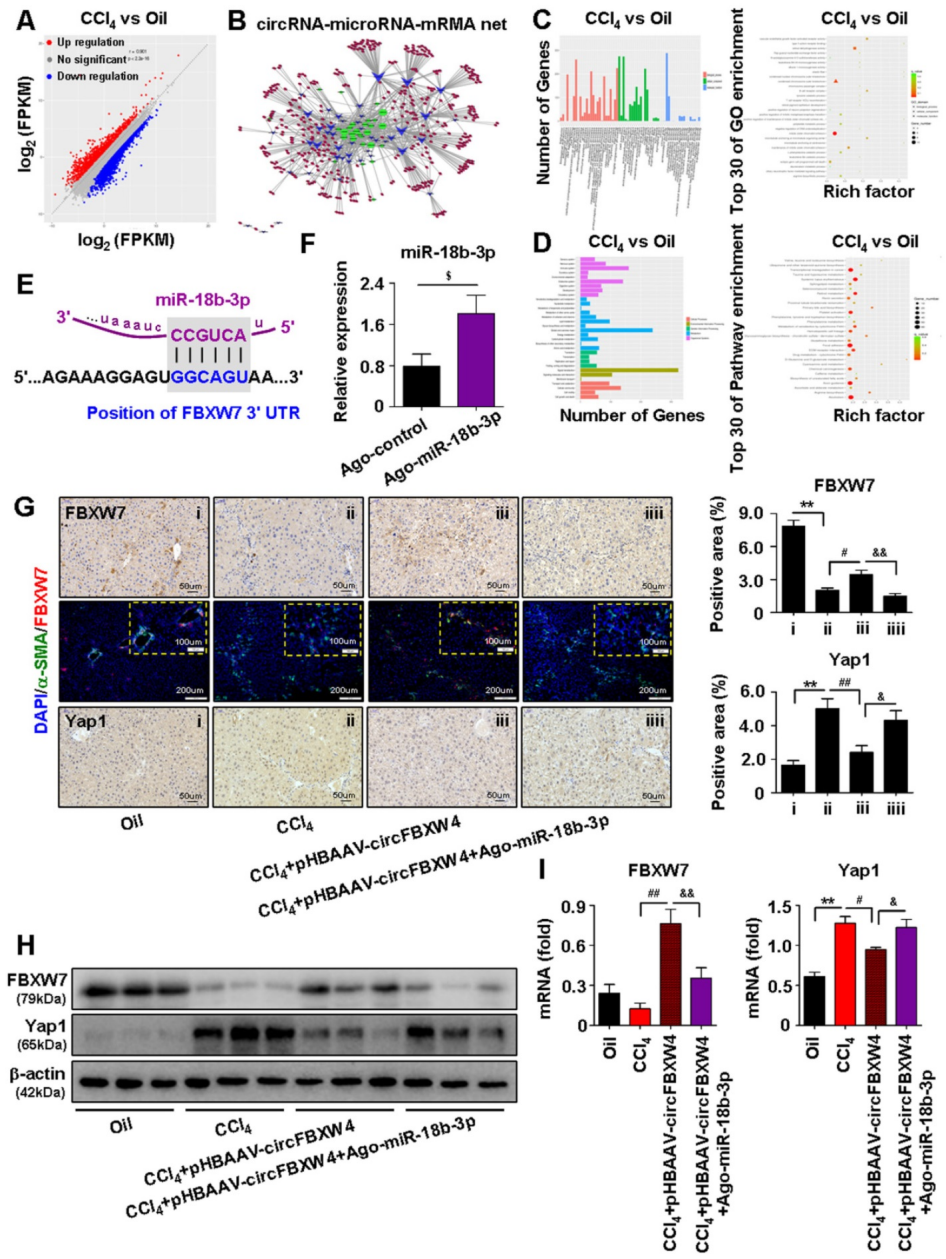
** Identification of circFBXW4/miR-18b-3p/FBXW7 axis. (A)** Scatter plots showed differentially expressed mRNAs in HSCs. **(B)** circRNAs-micorRNAs-mRNAs interaction network showed negative regulatory relationships combine the differentially expressed miRNAs with their differentially expressed circRNAs and mRNAs targets.** (C, D)** GO and KEGG enrichment analysis of differentially expressed genes in HSCs.** (E)** Binding sites of miR-18b-3p and 3'UTR of FBXW7.** (F)** Overexpression efficiency of miR-18b-3p in HSCs with miR-18b-3p-Up-agomir treatment. **(G)** Immunohistochemistry staining for FBXW7 and Yap1 in liver tissue, scale bar, 50 μM; Double-immunofluorescence staining showed co-localization of FBXW7 with α-SMA in liver tissue, nuclei were stained with DAPI, scale bar, 200 μM and 100 μM (up).** (H, I)** Protein expression and mRNA levels of FBXW7 and Yap1 in HSCs following circFBXW4 overexpression and miR-18b-3p-Up-agomir treatment. Data represent the mean ± s.e.m (n=6). ^$^*p*<0.05, ^$$^*p*<0.01 versus Control; *p<0.05, **p<0.01 versus Oil; ^#^*p*<0.05, ^##^*p*<0.01 versus CCl_4_; ^&^*p*<0.05, ^&&^*p*<0.01 versus CCl_4_+pHBAAV-circFBXW4.

MiRNAs play a central role in the ceRNA mechanism via binding to the 3'UTR or 5'UTR of target mRNAs to repress translation and induce degradation. To reveal target genes of circFBXW4/miR-18b-3p relate to HF, mRNAs expression profile in HSCs was analyzed by whole-transcriptome-seq (File S7-8). Differentially expressed mRNAs (DEMs) between HF and vehicle mice were showed in scatter plots (Figure 8A), followed by the DEMs GO and KEGG enrichment pathway analysis (Figure 8C-D). Next, an interaction network of circRNAs-miRNAs-mRNAs was established based on the negative regulatory relationship between differentially expressed miRNAs and their differentially expressed target circRNAs and mRNAs (Figure 8B). We found that FBXW7 is one of the target genes of miR-18b-3p, and expression of FBXW7 also reduced in HSCs from HF mice compared with vehicle by whole-transcriptome seq analysis (File S7). Binding sites of FBXW7 were identified within the miR-18b-3p sequences (Figure 8E). Importantly, overexpression of FBXW7 induces growth inhibition by increasing classical ferroptotic events, a form of programmed cell death recently discovered, and contributes to the autophagic degradation function in HSCs 33. Additionally, FBXW7 is recognized as a tumor suppressor in many cancer types, FBXW7 is responsible for the degradation of its target proteins, including yes associated protein-1 (Yap1) 34, c-Myc 35 and Notch1 36. Consistently, our results showed that expression of FBXW7 was decreased in both CCl_4_ induced and BDL operated HF mice (Figure 8H and Figure S1G). Next, we inhibited FBXW7 signaling by knockdown of FBXW7 (Figure S6A-B), results showed that treatment of siRNA-FBXW7 further promoted the levels of fibrogenic genes PDGF and Fibronectin in LX-2 cells (Figure S6C). In contrast, overexpression of FBXW7 suppressed fibrogenic genes levels in LX-2 cells transfected with FBXW7 plasmid (Figure S6D-F).

We further revealed that circFBXW4 exerted anti-fibrotic effects by sponging miR-18b-3p to regulate FBXW7. We elevated the levels of miR-18b-3p in mice by miR-Up-agomir delivery, and the overexpression efficiency of miR-18b-3p was confirmed (Figure 8F). Enhancing miR-18b-3p significantly increased the fibrogenic factors α-SMA, PDGF and TIMP-1 expression in HSCs (Figure S6G). Mechanistically, both immunostaining of FBXW7 in liver tissues and expression of FBXW7 in HSCs were consistently increased following circFBXW4 overexpression. However, the effect of pHBAAV-circFBXW4 treatment on FBXW7 was reversed following miR-18b-3p level upregulation (Figure 8G-I). In contrast, Yap1, a downstream of FBXW7, was oppositely regulated with indicated treatment (Figure 8G-I). Additionally, we found the reduction of cyclinE1, c-Myc, TIMP-1 and promotion of p21, MMP2 in HSCs following circFBXW4 overexpression, while the effects of pHBAAV-circFBXW4 treatment in HSCs were abolished after co-treatment with miR-18b-3p agomir (Figure S6H-I). These results demonstrated that circFBXW4 acted as a sponge of miR-18b-3p to eliminate the effects of miR-18b-3p on HF through circFBXW4/miR-18b-3p/FBXW7 axis.

## Discussion

### Construction of a circRNAs-miRNAs-mRNAs network in HSCs

Recently, circRNA has become a hot topic with rapidly advancing research on various diseases, however, function of majority of identified circRNAs remains elusive. HF is characterized by excessive ECM accumulation and progressive inflammation 37. HSCs are the major collagen-synthesizing cells following persistent liver injury 38, 39, liver macrophages are a driver of inflammatory responses and induce activation of HSCs to trigger liver fibrogenesis 31. Interestingly, HF is a reversible pathology after removal of the original source of damages 9, and our previous work confirmed that cessation of liver injury results in HF regression 40. To date, only a few circRNAs have been reported in HF, including the regulation of hsa_circ_0071410 on irradiated HSCs *in vitro* 24, circRNA-0067835 participated in Tβ4-depleted HSCs *in vitro* 25, and mmu_circ_34116 dysregulated in fibrotic liver tissue. circRNAs are tissue or developmental stage specific biomarkers. However, expression profile and function of circRNAs involved in HF remain largely unexplored. In this study, we identified the expression patterns of circRNAs, miRNAs and mRNAs in HSCs to find potential biomarkers relate to HF (Figure S8). We constructed a ceRNA network based on the correlation among circRNAs, miRNAs and mRNAs. Notably, we focused on the circRNA and miRNA candidates with similar expression pattern in vehicle and HF recovery mice, which opposite to HF mice, these candidates may promising relate to HF progression and regression stages.

### Expression pattern and function of circFBXW4 in HF

circRNA-seq revealed that circFBXW4 significantly downregulated in HF mice compared with vehicle, while the levels of circFBXW4 restored in HF recovery mice. Consistently, circFBXW4 decreased in HF patients compared with healthy controls. Dysregulation of circFBXW4 in HF promoted us to investigate circFBXW4 functional roles. First, we determined the characteristic and stability of circFBXW4, circFBXW4 derived from the FBXW4 gene, which relates to the developmental processes through the specific ubiquitination and subsequent proteolysis of target proteins 41. FBXW4 mutated and lost in human cancers, it acted as a tumor suppressor in pathophysiological processes. In this study, overexpression of circFBXW4 significantly inhibited HSCs activation, decreased myofibroblast transdifferentiation, attenuated liver fibrogenesis injury, reduced collagen deposition and suppressed the expression of fibrogenic factors. Furthermore, our findings revealed that circFBXW4 protected against inflammatory responses, reduced liver macrophage infiltration and suppressed the levels of pro-inflammatory cytokines. Interestingly, circFBXW4 increased the proportion of M2 macrophages, elevated the levels of the M2 macrophage marker IL-10, promoted the expression of IL-10R in HSCs. Indicating that circFBXW4 inhibited inflammation in HF, contributed to suppressing the immune-mediated activation of HSCs and the initiation of liver fibrogenesis. Taken together, these findings suggest anti-fibrotic effects of circFBXW4 in HF, circFBXW4 may serve as a potential biomarker for HF therapy.

### Identification of the circFBXW4/miR-18b-3p/FBXW7 axis in HSCs

circRNAs, contain multiple miRNA binding sites or miRNA response elements 15, act as miRNA sponges. Based on miRNA-mediated mRNA cleavage, circRNAs substantially regulate target gene expression. To assess miRNA candidates potentially associated with circFBXW4 and relate to HF, we selected miRNAs that targeted by circFBXW4 and dysregulated in liver fibrogenesis. Mechanistically, circFBXW4 function as a modulator by sponging miRNAs. It has been reported that increased expression of miRNA-18b relates to HCC and chronic hepatitis 42. In particular, we confirmed that miR-18b-3p increased in HSCs during liver fibrogenesis and expression pattern of miR-18b-3p was opposite to circFBXW4. Furthermore, overexpression of circFBXW4 directly reduced miR-18b-3p levels in HSCs. Additionally, we verified that miR-18b-3p binds to the 3'UTR of FBXW7, decreased expression of FBXW7 was elevated in liver fibrogenesis following circFBXW4 overexpression. However, effects of circFBXW4 on FBXW7 were partially abrogated after enhancing miR-18b-3p levels.

Collectively, this study revealed a novel regulatory axis formed by circFBXW4/miR-18b-3p/FBXW7 in HF. We investigated the expression pattern, function and mechanism of circFBXW4 in HF. We further confirmed expression of circFBXW4 rescued in HF regression stage, while the mechanism has not been clearly elucidated and further validation is required. Moreover, circFBXW4 provides a platform for harboring variety of liver or fibrosis related miRNA candidates, the involvement of circFBXW4 in ceRNA mechanisms still need to be addressed.

## Methods

### Animal studies

Mouse HF and HF recovery model were established as previously described 39. Littermate C57BL/6J mice (male, 8-week-old) were used in this study, mice were allocated to each group randomly, food and water were freely available throughout this study. Mouse HF model was induced by CCl_4_ intraperitoneal injections (1 ml/kg, CCl_4_ dissolved in olive oil at a ratio of 1 : 4), biweekly for 4 weeks. Vehicle mice received the same volume of olive oil only. Vehicle and HF mice were sacrificed three days after the final CCl_4_ injection. Mouse HF recovery model was established via 4 weeks CCl_4_ intraperitoneal injections then withdrawed the repeated liver damages, followed by 6 weeks' normal feeding and recovered spontaneously. Mouse acute toxic liver injury model was established by one intraperitoneal injection of CCl_4_ (1 ml/kg, CCl_4_ dissolved in olive oil at a ratio of 1 : 4), mice were sacrificed 24 h after CCl_4_ injection. Cholestatic hepatic fibrosis model was established according to previously described procedure 31. Briefly, after laparotomy, BDL group mice (male, 12-week-old) were established by common bile duct ligation, operated by double ligation using non-resorbable surgical sutures. Sham-operated mice were exposed to the same surgical procedures without ligation. Animals were sacrificed following 15-day period cholestasis and associated fibrosis development. All animal procedures were approved by the Animal Experimentation Ethics Committee of Anhui Medical University.

### Analysis of circRNA expression profile

#### RNA extraction and quality control

A MiRNeasy Mini Kit (Qiagen, Germany) was used to prepare total RNA from HSCs. Then, RNA was purified using RNA Clean XP Kit (Beckman Coulter, USA) and RNase-Free DNase Set (Qiagen, Germany). Next, quantity and integrity of RNA were detected by NanoDrop 2000 (Thermo Fisher Scientific, MA) and Agilent Bioanalyzer 2100 (Agilent technologies, USA), respectively.

#### Library preparation and high-throughput sequencing

TruSeq® Stranded Total RNA Sample Preparation kit (Illumina, USA) was used to the construction of RNA-seq libraries following the manufacturer's instructions. Then, RNA-seq libraries were quantified by Qubit® 2.0 Fluorometer (Life Technologies, USA), besides, RNA-seq libraries were validated by Agilent 2100 bioanalyzer (Agilent Technologies, USA). The insert size and the mole concentration were first confirmed, cBot was then used for generating the cluster with the library diluted to 10 pM and were sequenced in the Illumina HiSeq 2500 system (Illumina, USA). Construction and validation of the libraries and sequencing were performed at Origin Biotech (Shanghai, China).

#### Data Analysis

FastQC1 software (v. 0.11.3) was used to detect the quality control of RNA-Seq reads. With the exception of known Illumina TruSeq adapter sequences, poor reads and ribosomal RNA reads, RNA sequences were first trimmed with seqtk2 software. Then, trimmed reads were mapped to mouse reference genome by BWA-MEM software (v. 2.0.4). Next, circRNAs were predicted using CIRI software, circRNAs were matched to the circBase and terms as known circRNAs, the counts were normalized by SRPBM. The trimmed reads were also aligned to the mouse reference genome by the Hisat2 (v. 2.0.4). Stringtie (v. 1.3.0) was performed for each gene count from trimmed reads. Gene counts were normalized by trimmed mean of *M*-values (TMM), and fragments per kilobase of transcript per million mapped reads (FPKM) in Perl script. Differentially expressed circRNAs (DECs) between three groups were analyzed using edgeR software. Primary inclusion criteria for DECs were a FC≥2. circRNAs-miRNAs interaction was predicted by analyzing significantly dysregulated circRNAs, in accordance with Origin Biotech's custom-built software, based on miRanda software.

### Microarray analysis

The Affymetrix GeneChip® miRNA 4.0 Array (Affymetrix, USA) was used following the manufacturer's protocol. FlashTagTM Biotin HSR Labeling Kit (Affymetrix, USA) was utilized for Poly(A) biotin labeling and hybridization. Next, a GeneChip Hybridization Wash and Stain Kit (Thermo Fisher, USA) was used to dye the array and pictures, and original data were obtained by scanning. Differentially expressed miRNAs (DEMs) between three groups were identified through volcano plot filtering and fold change filtering (selected at llog_2_FCl>1). The miRNAs targets prediction was performed by two databases TargetScan and miRanda, the common targets were obtained. Next, the intersection of target genes and DEMs were screened using Venny. We conducted pathway analysis, followed by enrichment analysis in accordance with the gene information from GO and KEGG pathways.

### Analysis of the ceRNA network (ceRNET)

The circRNAs-miRNAs-mRNAs ceRNET was constructed based on the negative regulatory relationship between differentially expressed miRNAs and their differentially expressed target mRNAs and circRNAs. An interaction ceRNET was constructed by Cytoscape.

### Human liver samples

All human liver samples were obtained from the First Affiliated Hospital of Anhui Medical University (Anhui, China) between March 2016 and June 2019. This study was approved by Biomedical Ethics Committee of Anhui Medical University. All patients and volunteers in this study were provided written informed consent. Fourteen hepatic fibrosis liver tissues were collected from patients caused by hepatitis B virus (HBV) infection and hepatitis C virus (HCV) infection who underwent liver biopsy. Ten normal liver tissue samples were obtained from transplant donors. Samples were immediately frozen in liquid nitrogen then stored at -80 °C, part of each tissue samples was fixed and embedded, subjected to pathological staining. The characteristics of patients were shown in Table S3.

### pHBAAV-mediated overexpression of circFBXW4 in mice

Luciferase-labelled specific liver tissue location of pHBAAV-circFBXW4 and vector were designed and synthesized by Hanbio Biotechnology (Shanghai, China). pHBAAV-circFBXW4 and vector (1×10^12^ vg/ml), diluted in saline, were injected into the tail vein of mice, respectively. One week later, mouse HF model was established for 4 weeks after pHBAAV administration. Mice exposed to pHBAAV delivery were anaesthetized, effect of pHBAAV-circFBXW4 on liver tissue location was confirmed using an IVIS Lumina III Imaging System (Caliper Life Sciences, USA). For miR-Up-agomir treated mice, one week after HF modeling, mice received tail vein injection of miR-18b-3p agomir or NC agomir (10 mmol/kg, 4 times injections) synthesized by Genepharma (Shanghai, China). Mice were sacrificed after indicated treatment, liver tissues were paraformaldehyde-fixed and paraffin-embedded or subjected to isolation of primary liver cells. RNA oligonucleotide sequences used were listed in Table S2. The procedure used for the animal treatment was showed in Figure S9.

### Isolation of primary liver cells

Primary HSCs and liver macrophages were isolated from mouse liver tissues by a two-step collagenase-pronase perfusion as previously described 43. Briefly, liver tissue was perfused and digested with pronase (Sigma-Aldrich, USA) and collagenase (Roche, Switzerland). Suspension of dispersed liver cells was layered by gradient centrifugation with 11.5% and 20% optiPrep (Axis- shield, Norway) according to manufacture protocols, respectively. Liver sinusoidal endothelial cells (LSEC) were removed from liver macrophages fraction by selective adherence, due to LSEC poorly attach culture plastic dish. Isolated primary HSCs were cultured in Dulbecco's modified Eagle's medium (Gibco, USA) supplemented with 15% fetal bovine serum (Gibco, USA) and 1% penicillin/streptomycin in a humidified atmosphere of 5% CO_2_ at 37 °C.

### Histology and immunohistochemistry

Paraformaldehyde-fixed, paraffin-embedded liver tissues were sectioned (4 μm) for H&E, sirius red and masson staining as described previously 39. For immunohistochemistry staining, sections were incubated with antibodies against α-SMA (Abcam, ab32575, UK), FBXW7 (Abcam, ab74054, UK), F4/80 (CST, #70076, MA), p21 (Abcam, ab188224, UK), YAP1 (CST, #14074, MA) using a microwave-based antigen retrieval technique 44. Slides were scanned by an automatic digital slide scanner (Pannoramic MIDI, 3DHISTECH, Hungary) and analysed by the CaseViewer software. The positive staining areas were measured by Ipwin32 software.

### Inflammatory cytokines Array

Levels of inflammatory cytokines were tested by a C-Series Mouse Inflammation Antibody Array (RayBiotech, USA) following manufacturer's instructions for the semi-quantitative detection of 40 mouse proteins in liver lysate. This array consists of antibodies spotted in duplicate onto membranes. After blocking the membrane, liver lysate was incubated with the membrane at 4 °C for 18 h. Then, the membrane was washed and subjected to biotinylated antibody incubated at room temperature (RT) for 2 h. Next, the membrane was incubated with 1×HRP-streptavidin at 4 °C for 12 h. Signals were visualized by chemiluminescence imaging detection system (Bio-Rad, CA). The intensity was determined by Image J software (NIH, Bethesda, USA).

### DNA sequencing

RNA was reverse-transcribed into cDNA using PrimeScript RT Master Mix (Takara, Japan). Polymerase chain reaction (PCR) was performed using 2×Taq Master Mix (Takara, Japan) according to the manufacturer's protocol. PCR products was identified by DNA sequencer (ABI3730XL, USA).

### Pull-down assay

Biotinylated circFBXW4 probe was specifically designed and synthesized for binding to the junction site of circFBXW4. HSCs were washed by PBS then lysed on ice, cell lysate was incubated with 3 μg biotinylated probe at RT for 4 h. Next, Pierce™ Streptavidin Magnetic Beads (Thermo Fisher Scientific, USA) were prepared, beads were incubated with RNase-free bovine serum albumin (stock 10 mg/ml) and 10 μl yeast transfer RNA (tRNA) (stock 10 mg/ml) in lysis buffer at 4 °C for 3 h, to reduce nonspecific binding in this study. Prepared beads were used to pull down biotin-coupled RNA complexes rotated at 4 °C overnight. The next day, after washing the beads with lysis buffer for 3 times, miRNAs were detected by qRT-PCR. Probe sequences used were listed in Table S1.

### Fluorescence* in situ* hybridization

*In situ* hybridization was performed using specific probes for circFBXW4 and miR-18b-3p, respectively. 5'CY3-labelled miR-18b-3p probe and 5'FAM-labelled probe crossed the splice junction against circFBXW4 were designed and synthesized. Hybridization assay was performed by Fluorescent in Situ Hybridization Kit (Genepharma, China) according to the manufacturer's protocol. Liver sections were processed and incubated with probes at 37 °C for 16 h. The nuclei were stained by DAPI. Signals were detected by an inverted fluorescence microscope (OLYMPUS IX83, Japan). Probe sequences used were listed in Table S1.

### Cell culture

LX-2 cells, a human immortalized HSC line, were cultured in Dulbecco's modified Eagle's medium (Gibco, USA) supplemented with 10% fetal bovine serum (Gibco, USA) and 1% penicillin/ streptomycin in a humidified atmosphere of 5% CO_2_ at 37 °C.

### Construction of stable circFBXW4-overexpressing LX-2 cells

Lentivirus circFBXW4 and vector were designed and constructed by Hanbio Biotechnology (Shanghai, China). LX-2 cells were transfected with Lentivirus circFBXW4 or vector (multiplicity of infection, MOI=10). 5 μg/ml Polybrene (Hanbio Biotechnology, Shanghai, China) was used to enhance transfection efficiency. After 48 h transfection, 2 μg/ml Puromycin (Solarbio, China) was added in culture medium to screen stable transfected cells for 1 week. Surviving cells were used as stable circFBXW4-overexpressing LX-2 cells. Overexpression efficiency of circFBXW4 was confirmed by qRT-PCR. Primers used were listed in Table S1.

### circFBXW4 knockdown

Small interfering RNAs (siRNAs) of circFBXW4 were synthesized to target the junction site of circFBXW4. LX-2 cells were transfected with si-circFBXW4 using Lipofectamine RNAiMax (Life Technologies, USA) according to the manufacturer's protocol. After 6 h transfection, the culture medium was replaced with fresh medium for additional 48 h incubation. Silencing efficiency of circFBXW4 was confirmed by qRT-PCR after transfection. Sequences of si-circFBXW4 used were listed in Table S1.

### RNA extraction and qRT-PCR

Total RNA was isolated using TRIzol reagent (Invitrogen, CA) according to the manufacturer's protocol. Concentration and quality of RNA were measured by NanoDrop 2000 (Thermo Fisher Scientific, MA), paired samples were adjusted to the similar concentration for used. Divergent primers were designed for circRNAs. qRT-PCR assay was performed using CFX96 RT-PCR system (Bio-Rad, CA) with SYBR Premix Ex Taq™ II (Takara, Japan). Primers used were listed in Table S1.

### Cytoplasmic and nuclear fractionation assay

Cytoplasmic and nuclear fractionation assay was performed using Cytoplasmic & Nuclear RNA Purification Kit (#NGB-21000, Norgen) following the manufacturer's instructions. Briefly, cytoplasmic fraction and nuclear fraction were separated by centrifugation (12000 × g for 10 min) after lysing on ice. The supernatant was collected as cytoplasmic RNA fraction, and retain the pellet containing the nuclear RNA fraction. Then, cytoplasmic RNA and nuclear RNA were adsorbed to the Mini Spin column, respectively. Next, RNA samples were washed 3 times then the purified RNA was eluted.

### Rnase R and Actinomycin D treatment

Rnase R (Epicentre Technologies, USA) was used to degrade linear RNA. RNA samples were split to two aliquots: one used for Rnase R digestion, another used for control treated with reaction buffer only. For Rnase R+ group, 2 ug total RNA was incubated with Rnase R (0.2 μl, 20 U/μl) and 10× reaction buffer (0.6 μl) at 37 °C for 20 min, for Rnase R- group, 2 ug total RNA was incubated with DEPC-treated water (0.2 μl) and 10× reaction buffer (0.6 μl) at 37 °C for 20 min. Amount of mRNA and circRNA exposed to Rnase R treatment was detected by qRT-PCR in CFX96 RT-PCR system (Bio-Rad, CA). Transcription of HSCs was blocked by 2 μg/mL actinomycin D (Sigma, USA) treatment for 4 h, 8 h, 16 h, 24 h, cells were harvested at different time points. The stability of circFBXW4 and mRNA of FBXW4 was analyzed by qRT-PCR.

### Luciferase reporter assay

circFBXW4 sequences contain the target sites for miRNA candidates were synthesized and cloned into pSI-Check2 reporter vector downstream to firefly luciferase (pSI-Check2-circFBXW4-wildtype), mutant version of circFBXW4 (pSI-Check2-circFBXW4-mutant) was also generated with the deletion of complementary sites, respectively. The reporter vector, miR-18b-3p mimics or negative control were co-transfected in HEK-293T cells using Lipofectamine 3000 (Invitrogen, CA). Activity of firefly and renilla luciferase were measured by Dual-Luciferase system (Promega, USA) according to the manufacturer's protocol and detected by GloMax Multi Jr (Promega, USA).

### Flow cytometry analysis

Liver macrophages were isolated from mice and analyzed by flow cytometry as previously described 32. Cells were incubated with PE conjugated Anti- F4/80 (BD Biosciences, USA) and FITC conjugated Anti-CD11b (BD Biosciences, USA), then detected by CytoFLEX flow cytometer (Beckman Coulter, USA), the data were analyzed by CytExpert software (Beckman Coulter, USA). For cell cycle analysis, transfected LX-2 cells were fixed in 70% ethanol at 4 °C for 18 h then incubated with PE. Cell cycle was detected by CytoFLEX flow cytometer (Beckman Coulter, USA). The ratio of cells in G_0_/G_1_ phase was counted and compared. Apoptosis analysis was detected by Annexin V-FITC/PE staining using FACS Calibur flow cytometer (BD Biosciences, USA), the data were analyzed by FlowJo software (TreeStar, USA).

### CCK-8 assay

The proliferation of LX-2 cells was tested by Cell Counting Kit-8 assay. Approximately 2×10^4^ transfected LX-2 cells were seeded in 96-well plate, CCK-8 solution (10μl) was added to each well at 12 h, 24 h, 36 h, 48 h, 60 h. After incubation at 37 °C for 2 h, the optical density value at 450 nm was measured using Automatic microplate reader (BioTek, USA).

### 5-Ethynyl-2′-deoxyuridine assay

We utilized BeyoClick™ EdU Cell Proliferation Kit (Beyotime, China) to detect DNA synthesis and cell proliferation. Transfected LX-2 cells were seeded in 96-well plate and incubated for 24 h. Then, cell culture medium was added with 10 μM EdU and cells were incubated for another 6 h. Next, cells were fixed with 4% paraformaldehyde at RT for 15 min, followed by 0.3% Triton X-100 to permeabilize cell. Nucleic acids were stained with Hoechst33342. Signals were detected using an inverted fluorescence microscope (OLYMPUS IX83, Japan).

### Western blotting

Western blotting was performed using RIPA lysis buffer as previously described 39. Equal amount of protein was subjected to SDS-PAGE electrophoresis separated, then transferred to PVDF membrane (Millipore Corporation, USA) followed by blocking. Primary antibodies used in this study including α-SMA (Abcam, ab32575, UK), FBXW7 (Abcam, ab74054, UK), cyclinE1 (Abcam, ab71535, UK), c-Myc (Abcam, ab32072, UK), p21 (Abcam, ab188224, UK), YAP1 (CST, #14074, MA), TIMP-1 (Abcam, ab38978, UK) collagens I (Bioss, bs10423R, China), MMP2 (Bioss, bs4605R, China), β-actin (Bioworld, BS6007M, USA). Bands were visualized by enhanced chemiluminescence detection system (Bio-Rad, CA) then quantified by Image J software (NIH, Bethesda, USA) and normalized to internal control β-actin.

### Statistical analysis

Data collected from this study were expressed as mean ± s.e.m and analyzed using one-way analysis of variance (ANOVA), followed by Newman-Keuls *post-hoc test* (Prism 5.0 GraphPad Software, Inc, San Diego, CA, USA).

## Supplementary Material

Supplementary figures and tables.

Supplementary file 1.

Supplementary file 2.

Supplementary file 3.

Supplementary file 4.

Supplementary file 5.

Supplementary file 6.

Supplementary file 7.

Supplementary file 8.

## Abbreviations

α-SMA: alpha smooth muscle actin
ALT: alanine aminotransferase
BDL: bile duct ligation
CeRNA: competitive endogenous RNA
CircRNAs: circular RNAs
CCl4: carbon tetrachloride
CCL2: C-C motif chemokine ligand 2
CCL9: C-C motif chemokine ligand 9
DEMs: differentially expressed mRNAs
ECM: extracellular matrix
FBXW4: F box and WD 40 domain containing protein 4
FBXW7: F box and WD 40 domain containing protein 7
HCC: hepatocellular carcinoma
HF: hepatic fibrosis
HSCs: hepatic stellate cells
HYP: hydroxyproline
IHC: immunohistochemistry
LncRNAs: long non-coding RNAs
MiRNAs: microRNAs
NcRNAs: noncoding RNAs
Seq: sequencing
TGF-β1: transforming growth factor-β1
TIMP-1: tissue inhibitor of metallopeptidase-1
