# Supplementary material for: Circular RNA circFBXW4 suppresses hepatic fibrosis via targeting the miR-18b-3p/FBXW7 axis
Source: Theranostics. 2020 Mar 26;10(11):4851–70. doi: 10.7150/thno.42423 (PMC7163456; doi:10.7150/thno.42423)
Supplement: Supplementary file 1 — Supplementary figures and tables. [file thnov10p4851s1.pdf]

Figure legends

Figure 1. circRNAs profiling in HSCs from vehicle, HF and HF recovery mice. (A) The procedure of mouse acute toxic liver injury model, HF model and HF recovery model used for animal. **(B)** Pathology observation of H&E and sirius red staining of liver tissues, scale bar, 100 μM; immunohistochemistry staining for α-SMA in liver tissues, scale bar, 100 μM; immunofluorescence staining of α-SMA and collagens I in liver tissues, nuclei were stained with DAPI, scale bar, 200 μM. The positive staining areas were measured by Ipwin32 software. **(C)** Protein expression of α-SMA and collagens I in HSCs detected by western blot. **(D)** mRNA levels of α-SMA, PDGF, TIMP-1 and collagens I in HSCs detected by qRT-PCR. **(E)** A heatmap showed differentially expressed circRNAs with same expression pattern in vehicle and HF recovery mice, which opposite to HF mice. **(F)** circRNA composition in terms of genes distribution. **(G, H)** The coverage and distribution of differentially expressed circRNAs on the mouse chromosomes. Data represent the mean ± s.e.m (n=8). $*p<0.05$, $**p<0.01$ versus Oil; $^{\#}p<0.05$, $^{\#\#}p<0.01$ versus CCl_4.

Figure 2. Expression of circFBXW4 decreased in HF and rescued in HF recovery mice. (A) Eight differentially expressed circRNAs confirmed by qRT-PCR in HSCs (n=10-12). **(B)** Pathology observation of H&E staining from acute toxic liver injury tissue, scale bar, 100 μM; immunohistochemistry staining for α-SMA, scale bar, 100 μM. The positive staining areas were measured by Ipwin32 software. **(C)** mRNA level

of TGF-β1 and **(D)** relative expression of circFBXW4 in HSCs (n=6). **(E)** Relative expression of circFBXW4 in HSCs and **(F)** in serum. **(G)** mRNA level of FBXW4 in HSCs. **(H)** Expression pattern of circFBXW4 in HF progression and regression stages. **(I)** Pathology observation of H&E and masson staining of human liver tissue, scale bar, 100 μM; immunohistochemistry staining for α-SMA, scale bar, 100 μM. The positive staining areas were measured by Ipwin32 software. **(J)** Relative expression of circFBXW4, α-SMA, TIMP-1 and collagens I in human liver tissue detected by qRT-PCR (Control, n=10; Fibrosis, n=14). Data represent the mean ± s.e.m. $^{*}p<0.05$, $^{**}p<0.01$ versus Oil; $^{\#}p<0.05$, $^{\#\#}p<0.01$ versus CCl_4; $^{\&}p<0.05$, $^{\&\&}p<0.01$ versus Control.

Figure 3. Characterizations of circFBXW4. (A) Schematic diagram showed the genomic location and back-splicing pattern of circFBXW4. **(B)** Sanger sequencing of circFBXW4, the arrow represents the "head-to-tail" splicing site. **(C)** Divergent primers amplified circFBXW4 from cDNA by PCR and an agarose gel electrophoresis, rather than from gDNA, GAPDH was used as a linear control (n=3). **(D)** circFBXW4, rather than linear FBXW4 or GAPDH, resisted to Rnase R digestion (n=6). **(E)** circFBXW4 from cDNA was detected with divergent primers even exposed to Rnase R digestion, the opposite result showed from gDNA (n=3). **(F)** Relative expression of circFBXW4 and linear FBXW4 in HSCs treated with ActD+ (actinomycin D) at the indicated time points (n=6). **(G)** Cytoplasmic and nuclear fractionation assay revealed that circFBXW4 mainly localized in the cytoplasm (n=4). Data represent the mean ±

s.e.m. $^{\&}p<0.05$, $^{\&\&}p<0.01$ versus Rnase R- group; $^{*}p<0.05$, $^{**}p<0.01$ versus time 0.

Figure 4. Effects of circFBXW4 overexpression on the activation, proliferation and apoptosis of LX-2 cells. (A) Stable overexpression efficiency of circFBXW4, and mRNA levels of FBXW4, TGF-β1, TIMP-1, α-SMA and collagens I detected by qRT-PCR. **(B)** Protein expression of α-SMA and collagens I in LX-2 cells (n=4). **(C)** Immunofluorescence staining for α-SMA, nuclei were stained with DAPI, scale bar, 50 μM. **(D)** Effect of circFBXW4 overexpression on the proliferation of LX-2 cells detected by CCK8 assay at the indicated time points (n=6). **(E)** Assessment of DNA synthesis using Edu assay in LX-2 cells, nuclei were stained with Hoechst33342, scale bar, 200μM. **(F)** Flow cytometry showed the cell cycle distribution in G_0/G_1 phase increased following overexpression of circFBXW4. **(G)** Overexpression of circFBXW4 induced apoptosis of LX-2 cells with indicated treatment. Data represent the mean ± s.e.m. $^{*}p<0.05$, $^{**}p<0.01$ versus Control; $^{\#}p<0.05$, $^{\#\#}p<0.01$ versus LV-vector.

Figure 5. Anti-fibrotic effects of circFBXW4 in HF mice. (A) Mouse *in vivo* imaging analysis showed luciferase-labelled pHBAAV-circFBXW4 was specific located in mouse liver tissue. **(B)** Overexpression efficiency of circFBXW4 in HSCs from mice following pHBAAV-circFBXW4 administration. **(C)** Pathology observation of H&E staining, sirius red staining and immunohistochemistry staining for α-SMA in liver tissue, scale bar, 100 μM. The positive staining areas were

measured by Ipwin32 software. **(D)** Protein expression of α-SMA and collagens I in HSCs from mice after circFBXW4 overexpression. **(E, F)** Test of HYP in mouse liver tissue and ALT in serum. **(G)** mRNA levels of TGF-β1, α-SMA, collagens I and TIMP-1 in HSCs from mice following pHBAAV-circFBXW4 treatment. Data represent the mean ± s.e.m (n=8). $^{\$}p<0.05$, $^{\$\$}p<0.01$ versus Saline; $^{*}p<0.05$, $^{**}p<0.01$ versus Oil; $^{\#}p<0.05$, $^{\#\#}p<0.01$ versus CCl_4.

Figure 6. Anti-inflammation of circFBXW4 in HF mice. **(A)** Expression pattern of circFBXW4 in liver macrophages from vehicle and HF mice. **(B)** pHBAAV-circFBXW4 successful elevated circFBXW4 level in liver macrophages. **(C)** Levels of pro-inflammatory cytokines TNF-α and IL-1β in serum detected by ELISA. **(D)** mRNA levels of CCL2 and CCL9 in liver macrophages (n=8). **(E)** The up panel is a schematic representation of mouse inflammation antibody array containing 40 inflammation antibodies, positive control, negative control and blank spots with duplicates. Array showed the expression of inflammatory factors in mouse liver lysates, fold changes of differentially expressed 5 inflammatory factors (CCL2, CD153, IL-12 p70, TNF-RII, CCL9) were showed in the down panel. **(F)** Immunohistochemistry staining for F4/80 in liver tissue, scale bar, 50 μM. The positive staining areas were measured by Ipwin32 software. **(G)** Flow cytometric analysis showed the reduction of $F4/80^{+}CD11b^{+}$ liver macrophages in HF mice with pHBAAV-circFBXW4 treatment (n=4). Data represent the mean ± s.e.m. $^{\$}p<0.05$, $^{\$\$}p<0.01$ versus Saline; $^{*}p<0.05$, $^{**}p<0.01$ versus Oil; $^{\#}p<0.05$, $^{\#\#}p<0.01$ versus CCl_4.

Figure 7. Microarray analysis of HSCs and circFBXW4 binds miR-18b-3p. (A) Heatmap of differentially expressed miRNAs in HSCs from vehicle, HF and HF recovery mice. **(B)** circRNA-miRNA network showed differentially expressed miRNAs with their circRNA targets. **(C)** Differentially expressed miRNAs combine with circFBXW4 predicted miRNA targets, and a schematic showed the binding sites of miRNAs bind to circFBXW4. **(D)** The renilla luciferase activity analysis of wild type or mutant circFBXW4 and miRNAs mimics or negative control co-transfected into HEK-293T cells, respectively. Data are presented as the ratio of renilla luciferase activity to firefly activity (n=3). **(E)** Biotinylated circFBXW4 probe could capture 3 miRNA candidates by RNA pull-down analysis (n=4). **(F)** Schematic of miR-18b-3p sites in circFBXW4 based on complementary sequences. **(G)** Co-localization of circFBXW4 with miR-18b-3p was determined in mouse liver tissue by FISH, nuclei were stained with DAPI, scale bar, 200 μM and 100 μM. **(H)** Level of miR-18b-3p was suppressed in HSCs following circFBXW4 overexpression (n=7). Data represent the mean ± s.e.m. $^{*}p<0.05$, $^{**}p<0.01$ versus Negative mimics+circFBXW4-wt; $^{\#}p<0.05$, $^{\#\#}p<0.01$ versus miRNA-mimics+circFBXW4-wt; $^{\&}p<0.05$, $^{\&\&}p<0.01$ versus circFBXW4 probe; $^{\$}p<0.05$, $^{\$\$}p<0.01$ versus Oil; $^{@}p<0.05$, $^{@@}p<0.01$ versus CCl_4.

Figure 8. Identification of circFBXW4/miR-18b-3p/FBXW7 axis. (A) Scatter plots showed differentially expressed mRNAs in HSCs. **(B)** circRNAs-micorRNAs-mRNAs interaction network showed negative regulatory

relationships combine the differentially expressed miRNAs with their differentially expressed circRNAs and mRNAs targets. **(C, D)** GO and KEGG enrichment analysis of differentially expressed genes in HSCs. **(E)** Binding sites of miR-18b-3p and 3'UTR of FBXW7. **(F)** Overexpression efficiency of miR-18b-3p in HSCs with miR-18b-3p-Up-agomir treatment. **(G)** Immunohistochemistry staining for FBXW7 and Yap1 in liver tissue, scale bar, 50 μM; Double-immunofluorescence staining showed co-localization of FBXW7 with α-SMA in liver tissue, nuclei were stained with DAPI, scale bar, 200 μM and 100 μM (up). **(H, I)** Protein expression and mRNA levels of FBXW7 and Yap1 in HSCs following circFBXW4 overexpression and miR-18b-3p-Up-agomir treatment. Data represent the mean ± s.e.m (n=6). $^{\$}p<0.05$, $^{\$\$}p<0.01$ versus Control; $^{*}p<0.05$, $^{**}p<0.01$ versus Oil; $^{\#}p<0.05$, $^{\#\#}p<0.01$ versus CCl_4; $^{\&}p<0.05$, $^{\&\&}p<0.01$ versus CCl_4+pHBAAV-circFBXW4.

Supplementary Figure legends

Supplementary Figure. 1. (A) Test of ALT, AST, ALP and HA in serum from vehicle, HF and HF recovery mice (n=10). **(B)** circFBXW4 was downregulated in the 6-weeks CCl_4 intoxication HF mice compared to vehicle (n=12). **(C)** Pathology observation of H&E and sirius red staining of liver tissues, scale bar, 50 μM; The positive staining areas were measured by Ipwin32 software. **(D)** circFBXW4 was attenuated in the BDL-operated mice for 15 days compared to sham mice. **(E)** Test of ALT and AST in serum. **(F)** mRNA levels of α-SMA, collagens I, TGF-β1, PDGF and TIMP-1 in

HSCs detected by qRT-PCR. **(G)** Protein expression of FBXW7 in HSCs detected by western blot (n=12). Data represent the mean ± s.e.m. $^{*}p<0.05$, $^{**}p<0.01$ versus Oil; $^{\#}p<0.05$, $^{\#\#}p<0.01$ versus CCl_4; $^{\$}p<0.05$, $^{\$\$}p<0.01$ versus sham mice.

Supplementary Figure. 2. Effects of circFBXW4 knockdown on the activation and proliferation of LX-2 cells. **(A)** Head-to-tail splicing of circFBXW4 in RT-PCR products of LX-2 cells detected by sanger sequencing, the arrow showed the junction site of circFBXW4. **(B, D)** circFBXW4 was resisted to Rnase R digestion and showed stability feature in response to ActD+ treatment in LX-2 cells. **(C)** FISH assay showed the localization of circFBXW4 in cytoplasmic, U6 and β-actin were used as positive control for nuclear and cytoplasmic fractions, respectively, scale bar, 20 μM. **(E)** Schematic illustration of 2 siRNAs of circFBXW4 was designed specially targets the back-splicing site of circFBXW4. **(F)** Knockdown efficiency of siRNAs on circFBXW4, and mRNA levels of FBXW4, α-SMA and TGF-β1 in si-circFBXW4 treated LX-2 cells. **(G)** Protein expression of α-SMA and collagens I in LX-2 cells with circFBXW4 knockdown. **(H)** Effect of circFBXW4 knockdown on the proliferation of LX-2 cells detected by CCK8 assay at the indicated time points. **(I)** Flow cytometry showed the cell cycle distribution in G_0/G_1 phase decreased following circFBXW4 knockdown (n=3). Data represent the mean ± s.e.m. $^{\&}p<0.05$, $^{\&\&}p<0.01$ versus Rnase R- group; $^{\gamma}p<0.05$, $^{\gamma\gamma}p<0.01$ versus time 0; $^{*}p<0.05$, $^{**}p<0.01$ si#1 versus Control; $^{\#}p<0.05$, $^{\#\#}p<0.01$ si#2 versus Control; $^{@}p<0.05$, $^{@@}p<0.01$ si#1 versus si-NC; $^{\$}p<0.05$, $^{\$\$}p<0.01$ si#2 versus si-NC.

Supplementary Figure. 3. (A-D) Overexpression efficiency of circFBXW4 in liver tissues, hepatocytes (HEP), hepatic stellate cells (HSCs) and macrophages (Mφ) isolated from livers. **(E)** Protein expression of Bax, Bcl-2, c-Myc and CyclinD1 in HSCs detected by western blot. Data represent the mean ± s.e.m (n=8). *p<0.05, **p<0.01 versus Oil; $^{\#}p$<0.05, $^{\#\#}p$<0.01 versus CCl_4.

Supplementary Figure. 4. circFBXW4 induces M2 macrophage phenotype in HF. **(A)** Double-immunofluorescence staining of M2 macrophages (F4/80^{+}/CD206^{+}), nuclei were stained with DAPI, scale bar, 100 μM. **(B)** mRNA level of IL-10 in liver macrophages and **(C)** circulating level of IL-10 in serum. **(D)** Level of IL-10R in HSCs. **(E)** mRNA levels of TGF-β1, α-SMA and TIMP-1 in HSCs with IL-10 stimulation detected by qRT-PCR. Data represent the mean ± s.e.m (n=6). *p<0.05, **p<0.01 versus Oil; $^{\#}p$<0.05, $^{\#\#}p$<0.01 versus CCl_4.

Supplementary Figure. 5. (A) Level of circFBXW4 was detected in different organs (heart, spleen, lung and kidney). **(B)** Quantification of positive staining areas in immunohistochemistry staining of α-SMA for Figure 5C, measured by ipwin32 software. **(C)** Quantification of positive staining areas in immunohistochemistry staining of F4/80 for Figure 6F, measured by ipwin32 software. **(D)** Effects of liver-specific circFBXW4 overexpression on pro-inflammatory cytokine IL-8 level and anti-inflammatory cytokine IL-10 level in heart tissue. **(E)** Effects of

liver-specific circFBXW4 overexpression on pro-inflammatory cytokines IL-1β level and IL-6 level in spleen tissue. **(F)** Effects of liver-specific circFBXW4 overexpression on pro-inflammatory cytokines TNF-α, IL-1β level and anti-inflammatory cytokine IL-10 level in lung tissue. **(G)** Effects of liver-specific circFBXW4 overexpression on pro-inflammatory cytokine IL-6 level in kidney tissue. Data represent the mean ± s.e.m (n=6). $^{*}p<0.05$, $^{**}p<0.01$ versus Oil; $^{\#}p<0.05$, $^{\#\#}p<0.01$ versus CCl_4.

Supplementary Figure. 6. (A, B) Protein expression and mRNA level of FBXW7 in LX-2 cells treated with FBXW7 siRNA transfection. **(C)** mRNA levels of PDGF and Fibronectin detected by qRT-PCR. **(D, E)** Protein expression and mRNA level of FBXW7 in LX-2 cells with FBXW7 plasmid transfection. **(F)** mRNA levels of PDGF and Fibronectin detected by qRT-PCR (n=4). **(G)** Levels of α-SMA, PDGF and TIMP-1 in HSCs following miR-18b-3p upregulation (n=6). **(H)** Protein expression of cyclinE1, c-Myc, TIMP1, MMP2 and p21 in HSCs with circFBXW4 overexpression and exposed to miR-18b-3p upregulation (n=6). **(I)** mRNA level of p21 in HSCs detected by qRT-PCR. $^{@}p<0.05$, $^{@@}p<0.01$ versus siRNA-NC; $^{\%}p<0.05$, $^{\%\%}p<0.01$ versus plasmid-NC; $^{\$}p<0.05$, $^{\$\$}p<0.01$ versus Ago-control; Data represent the mean ± s.e.m. $^{*}p<0.05$, $^{**}p<0.01$ versus Oil; $^{\#}p<0.05$, $^{\#\#}p<0.01$ versus CCl_4; $^{\&}p<0.05$, $^{\&\&}p<0.01$ versus CCl_4+pHBAAV-circFBXW4.

Supplementary Figure. 7. Quantification of bands in western blot analysis, measured

by Image J software.

Supplementary Figure. 8. Construction of a circRNA-miRNA-mRNA network in HSCs.

Supplementary Figure. 9. The procedure used for the animal treatment.

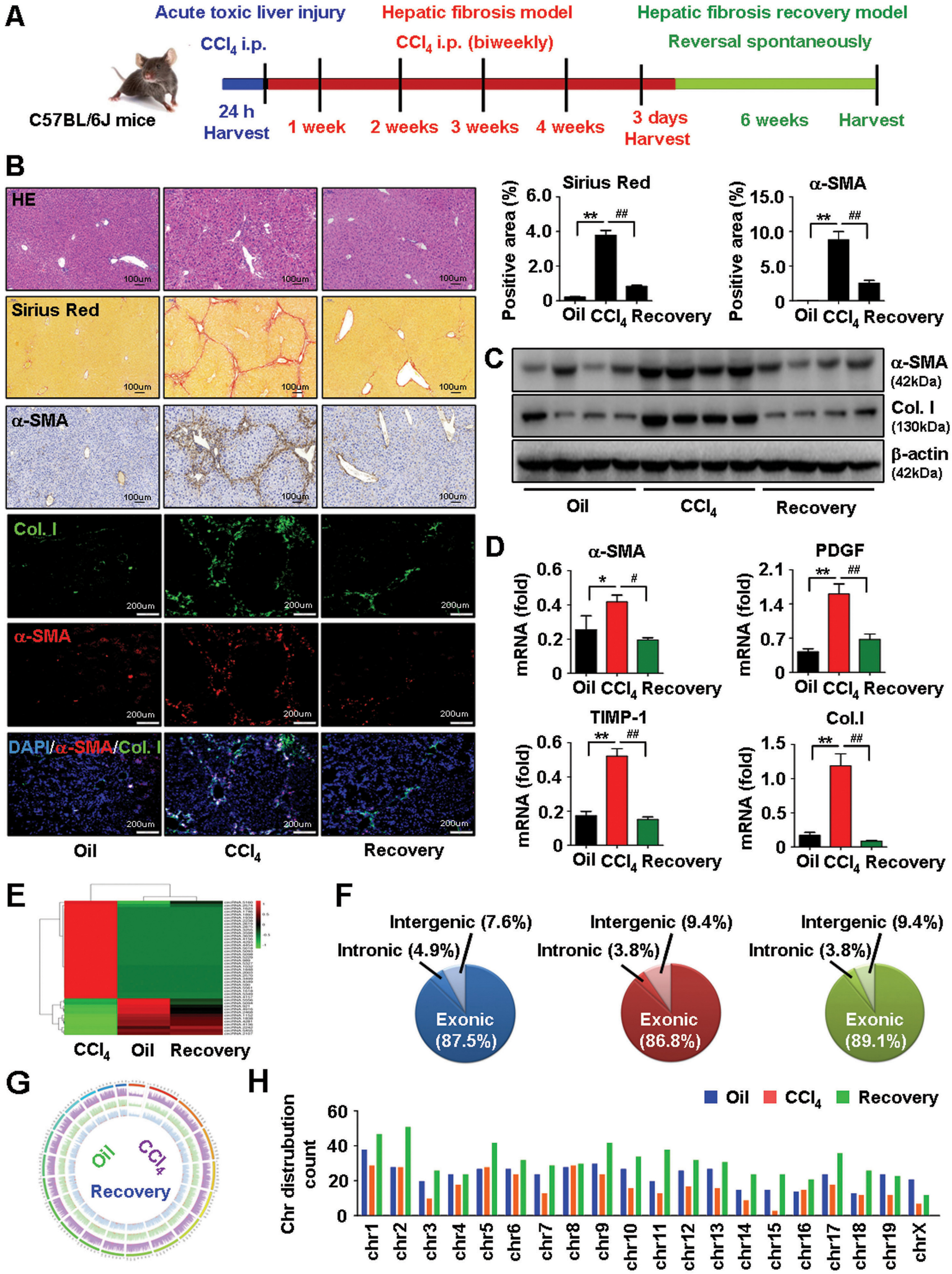

A
Acute toxic liver injury
Hepatic fibrosis model
Hepatic fibrosis recovery model
CCl4 i.p.
CCl4 i.p. (biweekly)
Reversal spontaneously
C57BL/6J mice
24 h Harvest
1 week
2 weeks
3 weeks
4 weeks
3 days Harvest
6 weeks
Harvest
B
HE
Sirius Red
α-SMA
Col. I
DAPI/α-SMA/Col. I
100um
200um
Oil
CCl4
Recovery
Sirius Red
α-SMA
Positive area (%)
C
α-SMA (42kDa)
Col. I (130kDa)
β-actin (42kDa)
D
α-SMA
PDGF
TIMP-1
Col.I
mRNA (fold)
E
F
Intergenic (7.6%)
Intronic (4.9%)
Exonic (87.5%)
Intergenic (9.4%)
Intronic (3.8%)
Exonic (86.8%)
Intergenic (9.4%)
Intronic (3.8%)
Exonic (89.1%)
G
H
Chr distrubution count
chr1
chr2
chr3
chr4
chr5
chr6
chr7
chr8
chr9
chr10
chr11
chr12
chr13
chr14
chr15
chr16
chr17
chr18
chr19
chrX

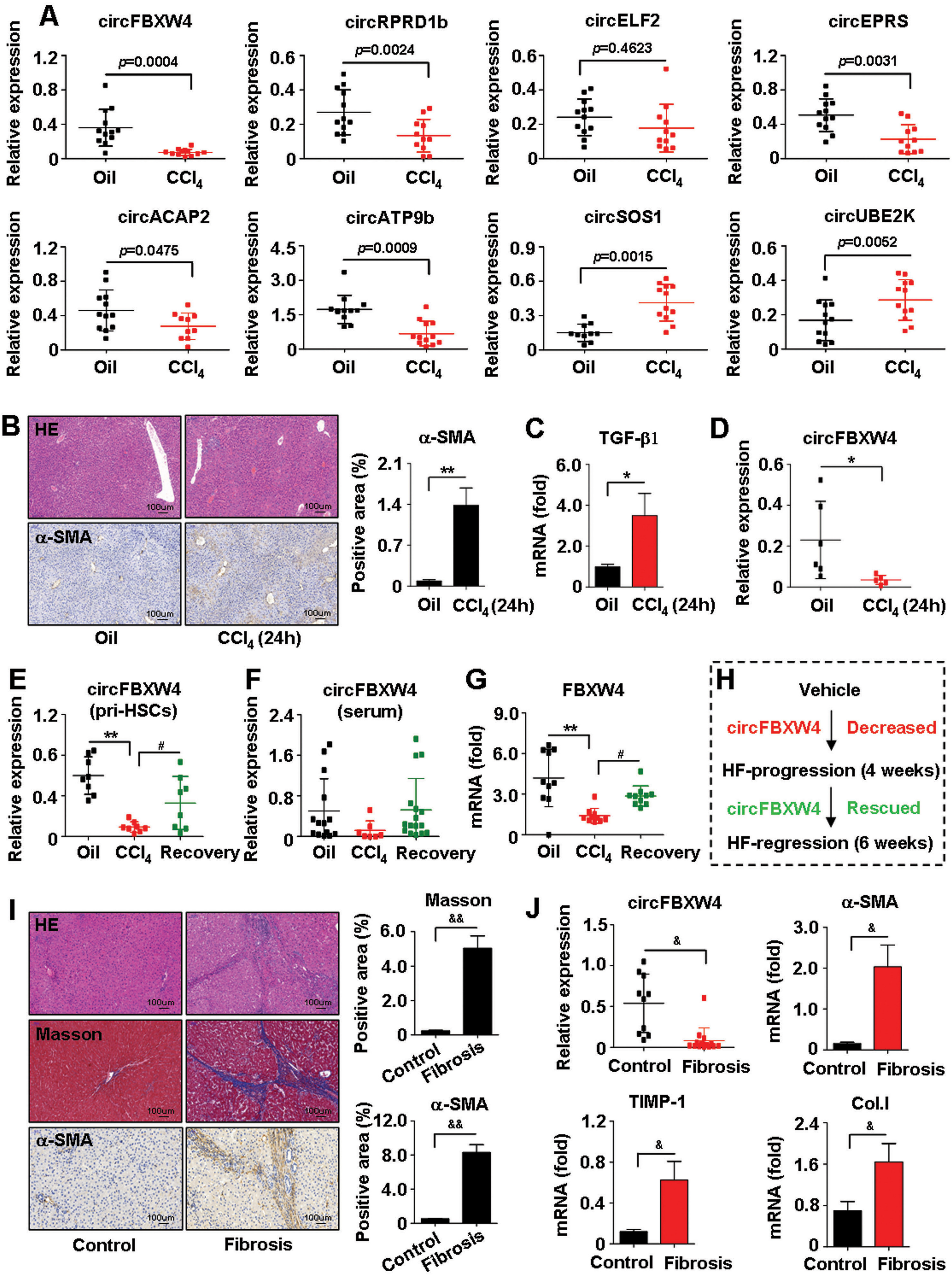
A
circFBXW4
p=0.0004
circRPRD1b
p=0.0024
circELF2
p=0.4623
circEPRS
p=0.0031
circACAP2
p=0.0475
circATP9b
p=0.0009
circSOS1
p=0.0015
circUBE2K
p=0.0052
Relative expression
Oil
CCl4
B
HE
α-SMA
Oil
CCl4 (24h)
100um
α-SMA
Positive area (%)
**
Oil
CCl4 (24h)
C
TGF-β1
mRNA (fold)
*
D
circFBXW4
Relative expression
*
E
circFBXW4
(pri-HSCs)
**
#
Oil
CCl4
Recovery
F
circFBXW4
(serum)
G
FBXW4
mRNA (fold)
H
Vehicle
circFBXW4
Decreased
HF-progression (4 weeks)
circFBXW4
Rescued
HF-regression (6 weeks)
I
HE
Masson
α-SMA
Control
Fibrosis
Masson
&&
Positive area (%)
J
circFBXW4
&
α-SMA
TIMP-1
Col.I
mRNA (fold)
Relative expression

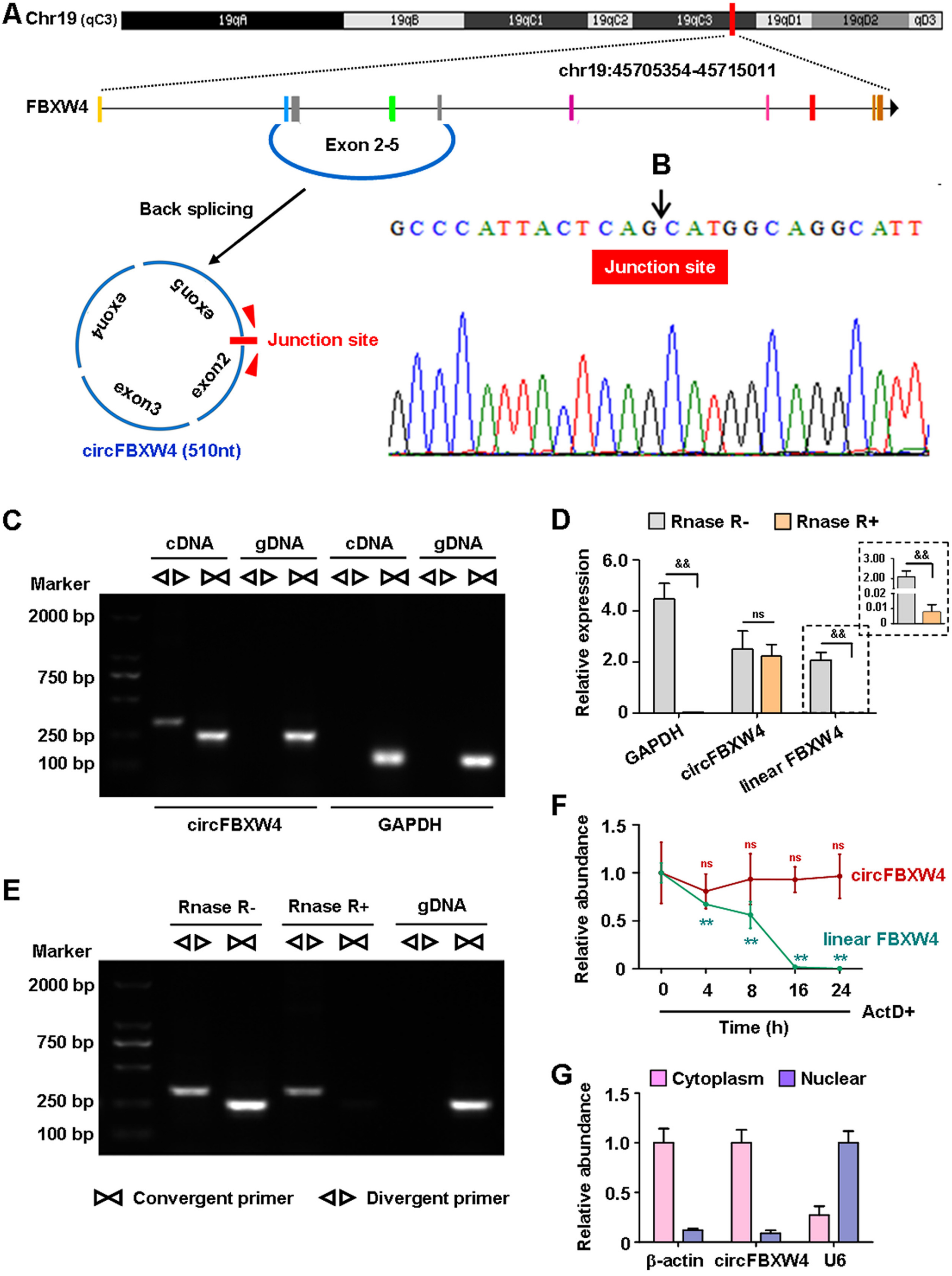

A
Chr19 (qC3)
19qA
19qB
19qC1
19qC2
19qC3
19qD1
19qD2
qD3
chr19:45705354-45715011
FBXW4
Exon 2-5
Back splicing
exon5
exon4
exon2
exon3
Junction site
circFBXW4 (510nt)
B
GCCCATTACTCAGCATGGCAGGCATT
Junction site
C
cDNA
gDNA
cDNA
gDNA
Marker
2000 bp
750 bp
250 bp
100 bp
circFBXW4
GAPDH
D
Rnase R-
Rnase R+
Relative expression
6.0
4.0
2.0
0
&&
ns
&&
3.00
2.00
0.02
0.01
0
&&
GAPDH
circFBXW4
linear FBXW4
E
Rnase R-
Rnase R+
gDNA
Marker
2000 bp
750 bp
250 bp
100 bp
Convergent primer
Divergent primer
F
Relative abundance
1.5
1.0
0.5
0
ns
ns
ns
ns
circFBXW4
**
**
**
**
linear FBXW4
0
4
8
16
24
ActD+
Time (h)
G
Cytoplasm
Nuclear
Relative abundance
1.5
1.0
0.5
0
β-actin
circFBXW4
U6

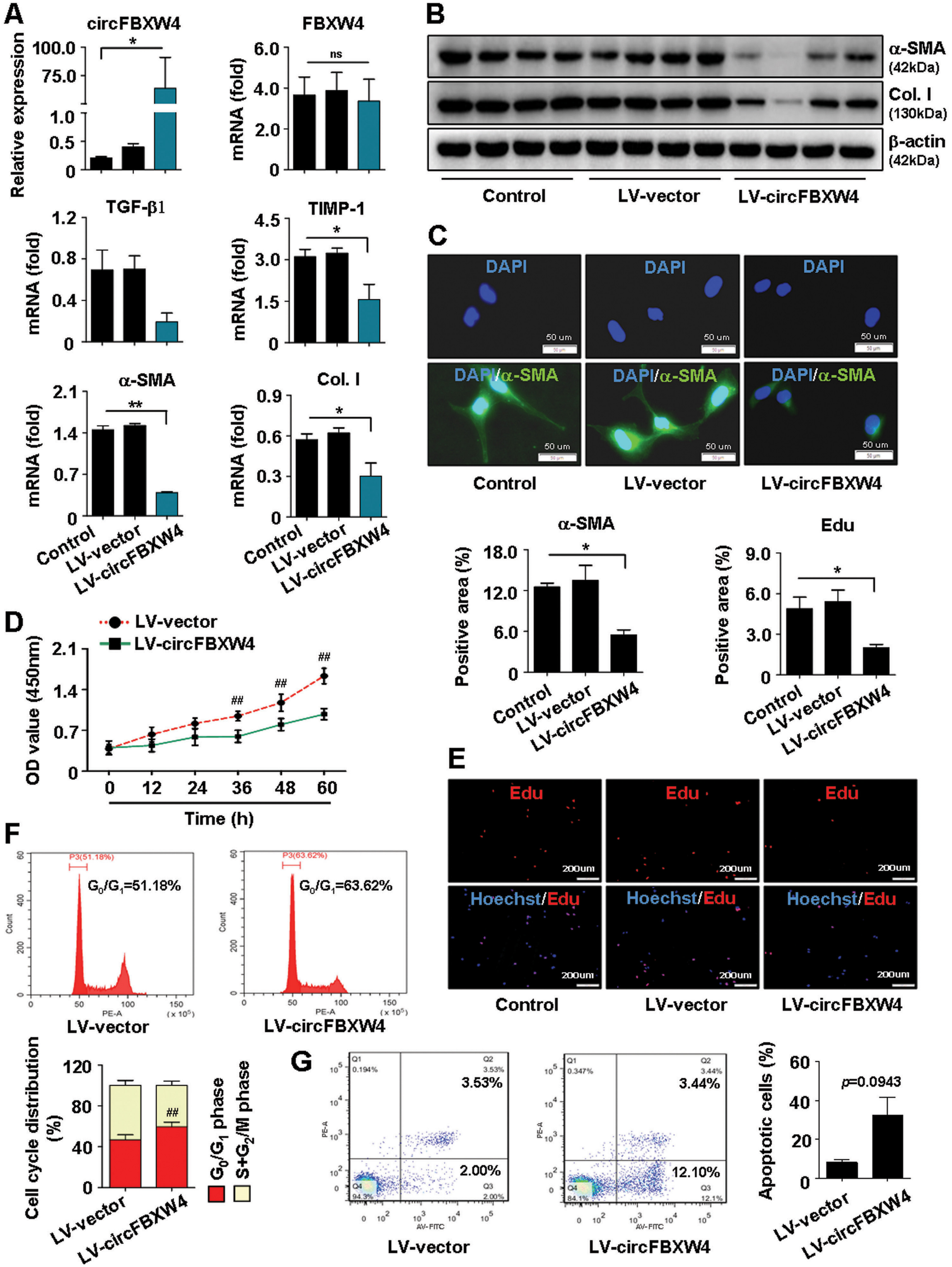
A
circFBXW4
FBXW4
Relative expression
mRNA (fold)
ns
TGF-β1
TIMP-1
α-SMA
Col. I
Control
LV-vector
LV-circFBXW4
B
α-SMA (42kDa)
Col. I (130kDa)
β-actin (42kDa)
Control
LV-vector
LV-circFBXW4
C
DAPI
DAPI/α-SMA
50 um
Control
LV-vector
LV-circFBXW4
α-SMA
Edu
Positive area (%)
D
LV-vector
LV-circFBXW4
OD value (450nm)
Time (h)
##
E
Edu
Hoechst/Edu
200um
Control
LV-vector
LV-circFBXW4
F
P3(51.18%)
G0/G1=51.18%
P3(63.62%)
G0/G1=63.62%
Count
PE-A
LV-vector
LV-circFBXW4
Cell cycle distribution (%)
G0/G1 phase
S+G2/M phase
G
3.53%
2.00%
3.44%
12.10%
AV-FITC
LV-vector
LV-circFBXW4
Apoptotic cells (%)
p=0.0943

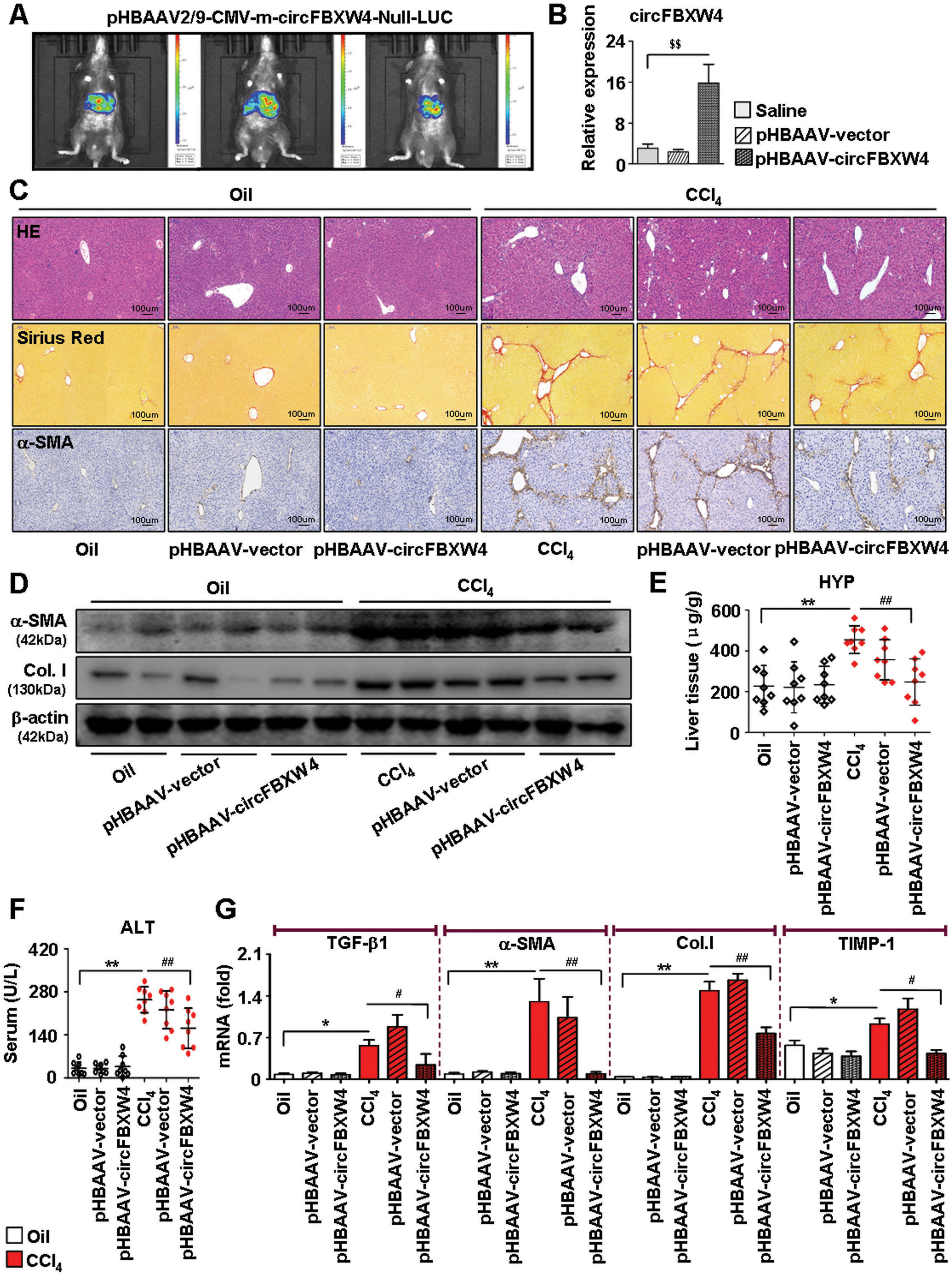

A
pHBAAV2/9-CMV-m-circFBXW4-Null-LUC
B
circFBXW4
Relative expression
$$
Saline
pHBAAV-vector
pHBAAV-circFBXW4
C
Oil
CCl4
HE
Sirius Red
α-SMA
100um
Oil
pHBAAV-vector
pHBAAV-circFBXW4
CCl4
pHBAAV-vector
pHBAAV-circFBXW4
D
Oil
CCl4
α-SMA (42kDa)
Col. I (130kDa)
β-actin (42kDa)
Oil
pHBAAV-vector
pHBAAV-circFBXW4
CCl4
pHBAAV-vector
pHBAAV-circFBXW4
E
HYP
Liver tissue (μ g/g)
**
##
Oil
pHBAAV-vector
pHBAAV-circFBXW4
CCl4
pHBAAV-vector
pHBAAV-circFBXW4
F
ALT
Serum (U/L)
**
##
Oil
pHBAAV-vector
pHBAAV-circFBXW4
CCl4
pHBAAV-vector
pHBAAV-circFBXW4
Oil
CCl4
G
TGF-β1
α-SMA
Col.I
TIMP-1
mRNA (fold)
*
#
**
##
**
##
*
#
Oil
pHBAAV-vector
pHBAAV-circFBXW4
CCl4
pHBAAV-vector
pHBAAV-circFBXW4

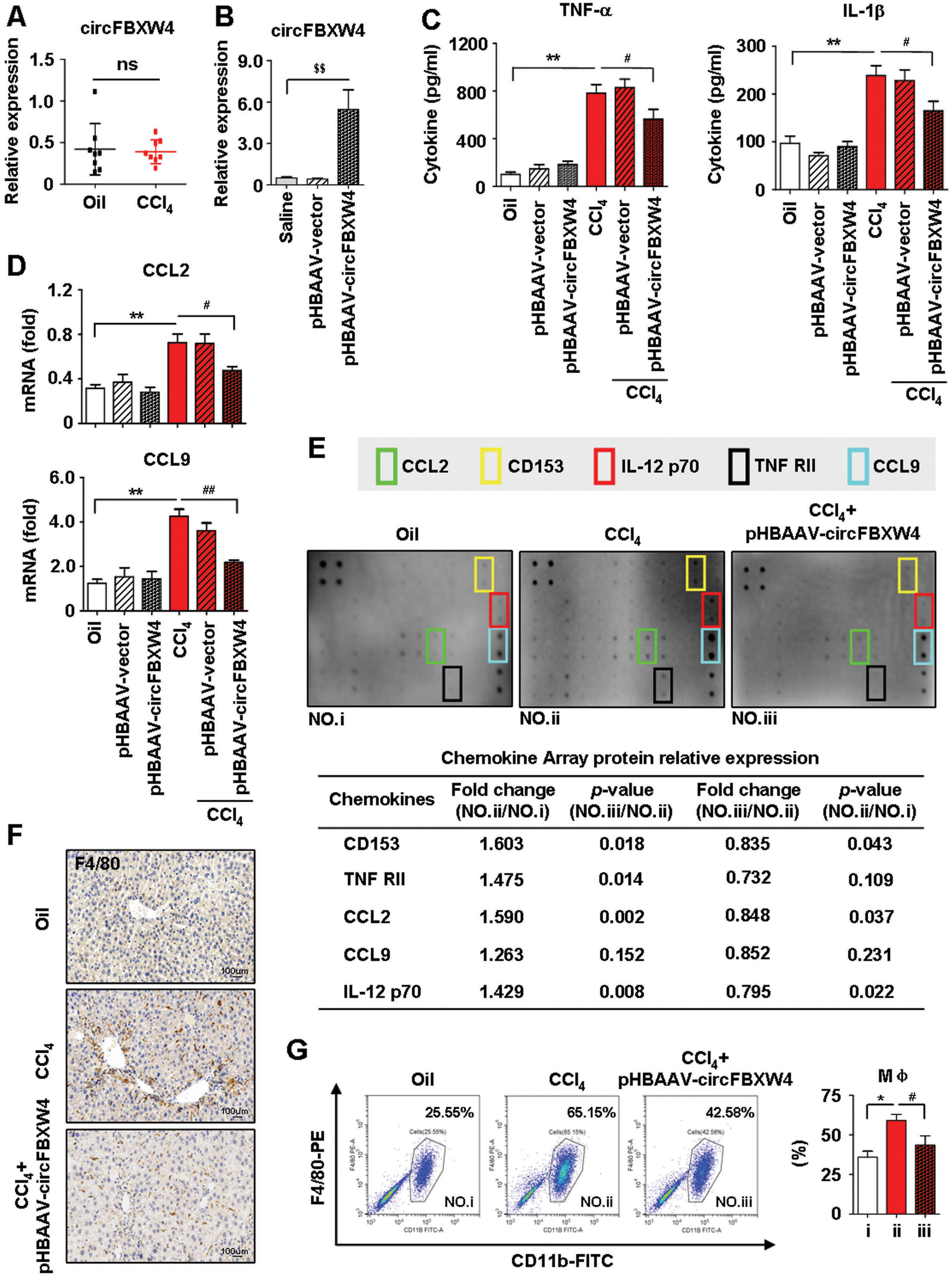

Chemokine Array protein relative expression

Chemokines	Fold change (NO.ii/NO.i)	*p*-value (NO.iii/NO.ii)	Fold change (NO.iii/NO.ii)	*p*-value (NO.ii/NO.i)
CD153	1.603	0.018	0.835	0.043
TNF RII	1.475	0.014	0.732	0.109
CCL2	1.590	0.002	0.848	0.037
CCL9	1.263	0.152	0.852	0.231
IL-12 p70	1.429	0.008	0.795	0.022

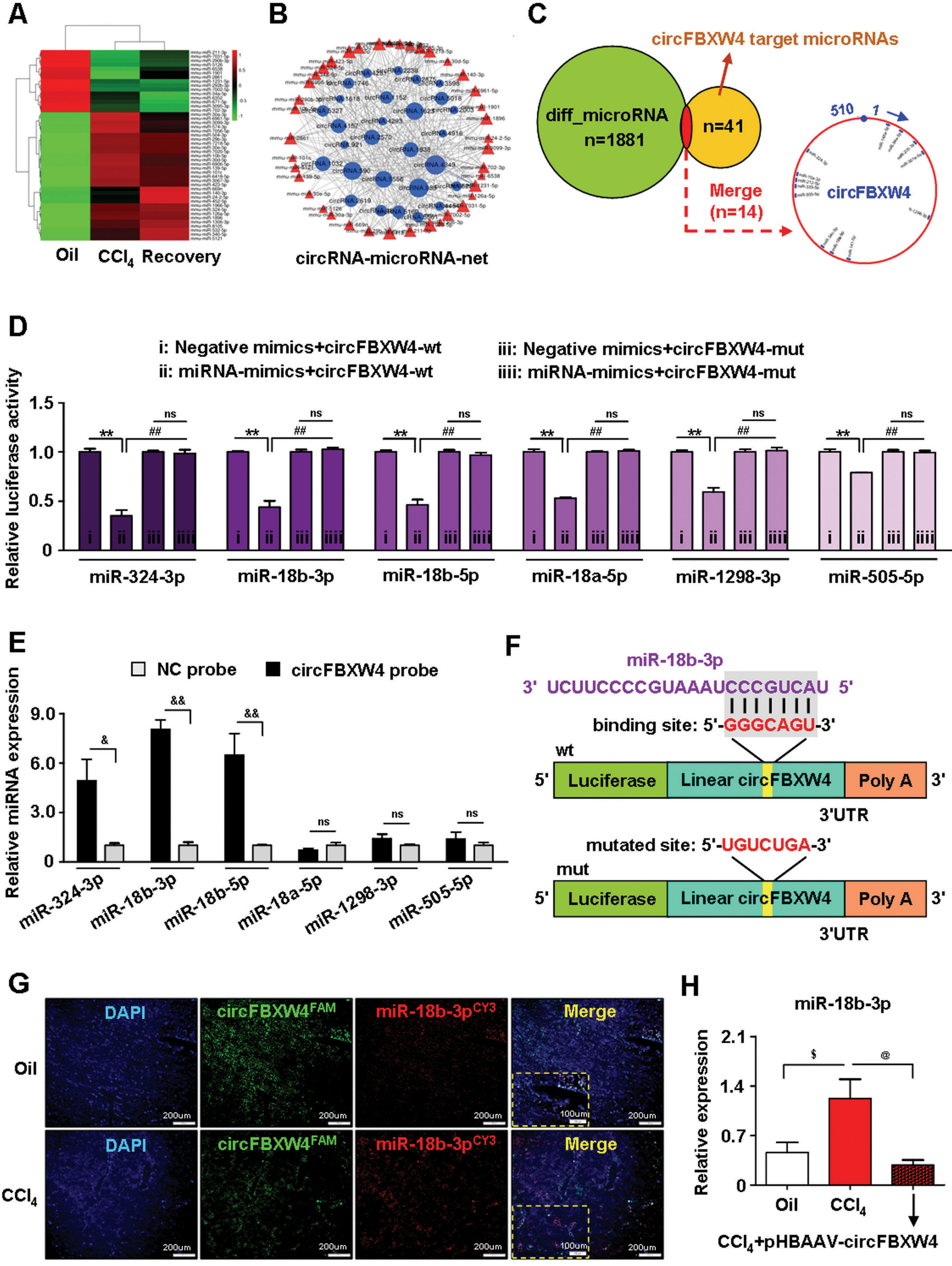

A
B
C
D
E
F
G
H
Oil
CCl4
Recovery
circRNA-microRNA-net
circFBXW4 target microRNAs
diff_microRNA
n=1881
n=41
Merge
(n=14)
510
1
circFBXW4
i: Negative mimics+circFBXW4-wt
ii: miRNA-mimics+circFBXW4-wt
iii: Negative mimics+circFBXW4-mut
iiii: miRNA-mimics+circFBXW4-mut
Relative luciferase activity
1.5
1.0
0.5
0
**
##
ns
i
ii
iii
iiii
miR-324-3p
miR-18b-3p
miR-18b-5p
miR-18a-5p
miR-1298-3p
miR-505-5p
NC probe
circFBXW4 probe
Relative miRNA expression
9.0
6.0
3.0
0
&
&&
miR-18b-3p
3' UCUUCCCCGUAAAUCCCGUCAU 5'
binding site: 5'-GGGCAGU-3'
wt
5' Luciferase Linear circFBXW4 Poly A 3'
3'UTR
mutated site: 5'-UGUCUGA-3'
mut
DAPI
circFBXW4FAM
miR-18b-3pCY3
Merge
200um
100um
Relative expression
2.1
1.4
0.7
$
@
CCl4+pHBAAV-circFBXW4

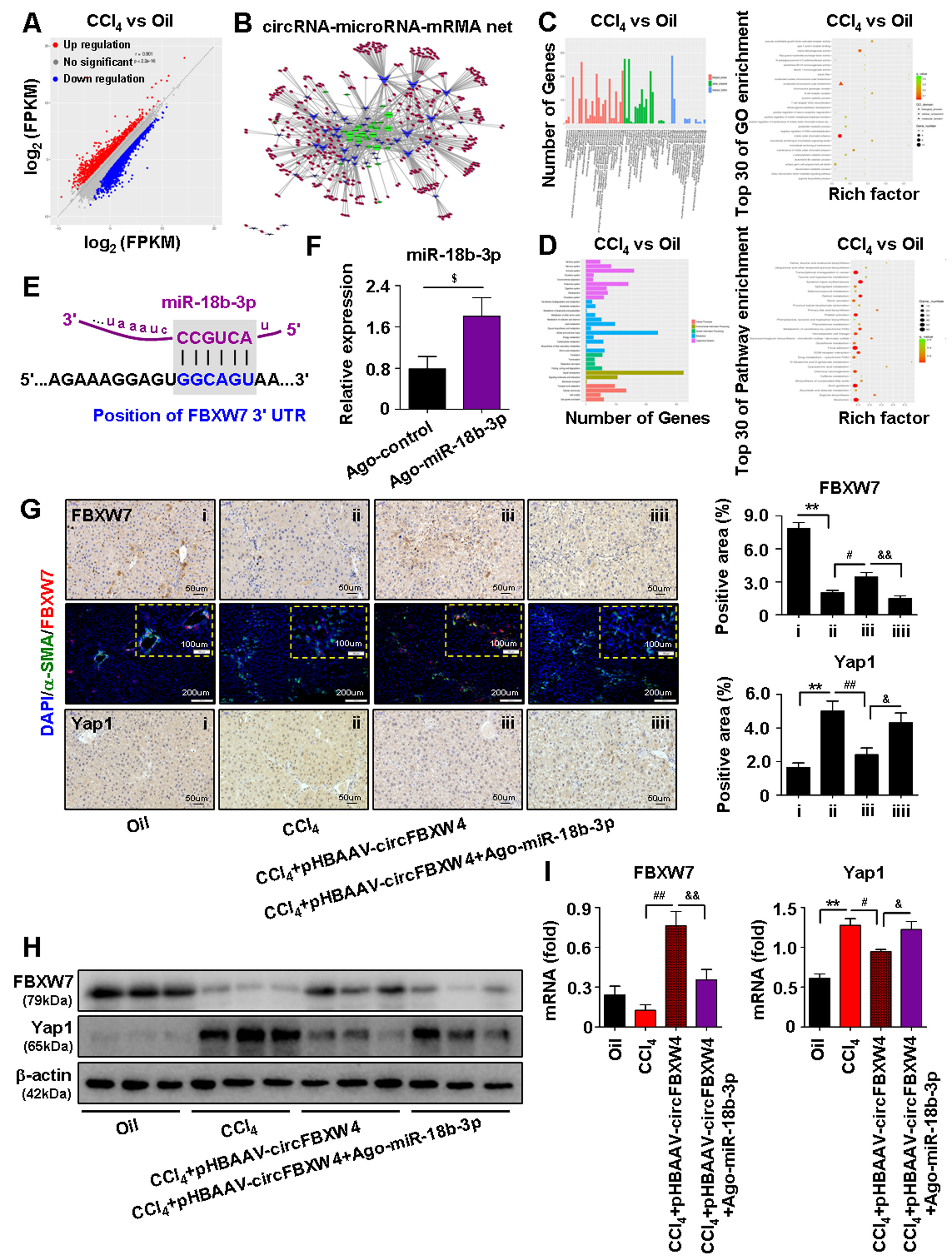

A
CCl4 vs Oil
Up regulation
No significant
Down regulation
log2 (FPKM)
log2 (FPKM)
B
circRNA-microRNA-mRMA net
C
CCl4 vs Oil
Number of Genes
Top 30 of GO enrichment
CCl4 vs Oil
Rich factor
D
CCl4 vs Oil
Number of Genes
Top 30 of Pathway enrichment
CCl4 vs Oil
Rich factor
E
miR-18b-3p
3' ...uaaauc CCGUCA u 5'
5'...AGAAAGGAGUGGCAGUAA...3'
Position of FBXW7 3' UTR
F
miR-18b-3p
Relative expression
$
Ago-control
Ago-miR-18b-3p
G
FBXW7
DAPI/α-SMA/FBXW7
Yap1
i
ii
iii
iiii
50um
100um
200um
Oil
CCl4
CCl4+pHBAAV-circFBXW4
CCl4+pHBAAV-circFBXW4+Ago-miR-18b-3p
FBXW7
Positive area (%)
Yap1
Positive area (%)
H
FBXW7 (79kDa)
Yap1 (65kDa)
β-actin (42kDa)
Oil
CCl4
CCl4+pHBAAV-circFBXW4
CCl4+pHBAAV-circFBXW4+Ago-miR-18b-3p
I
FBXW7
mRNA (fold)
Yap1
mRNA (fold)
Oil
CCl4
CCl4+pHBAAV-circFBXW4
CCl4+pHBAAV-circFBXW4 +Ago-miR-18b-3p

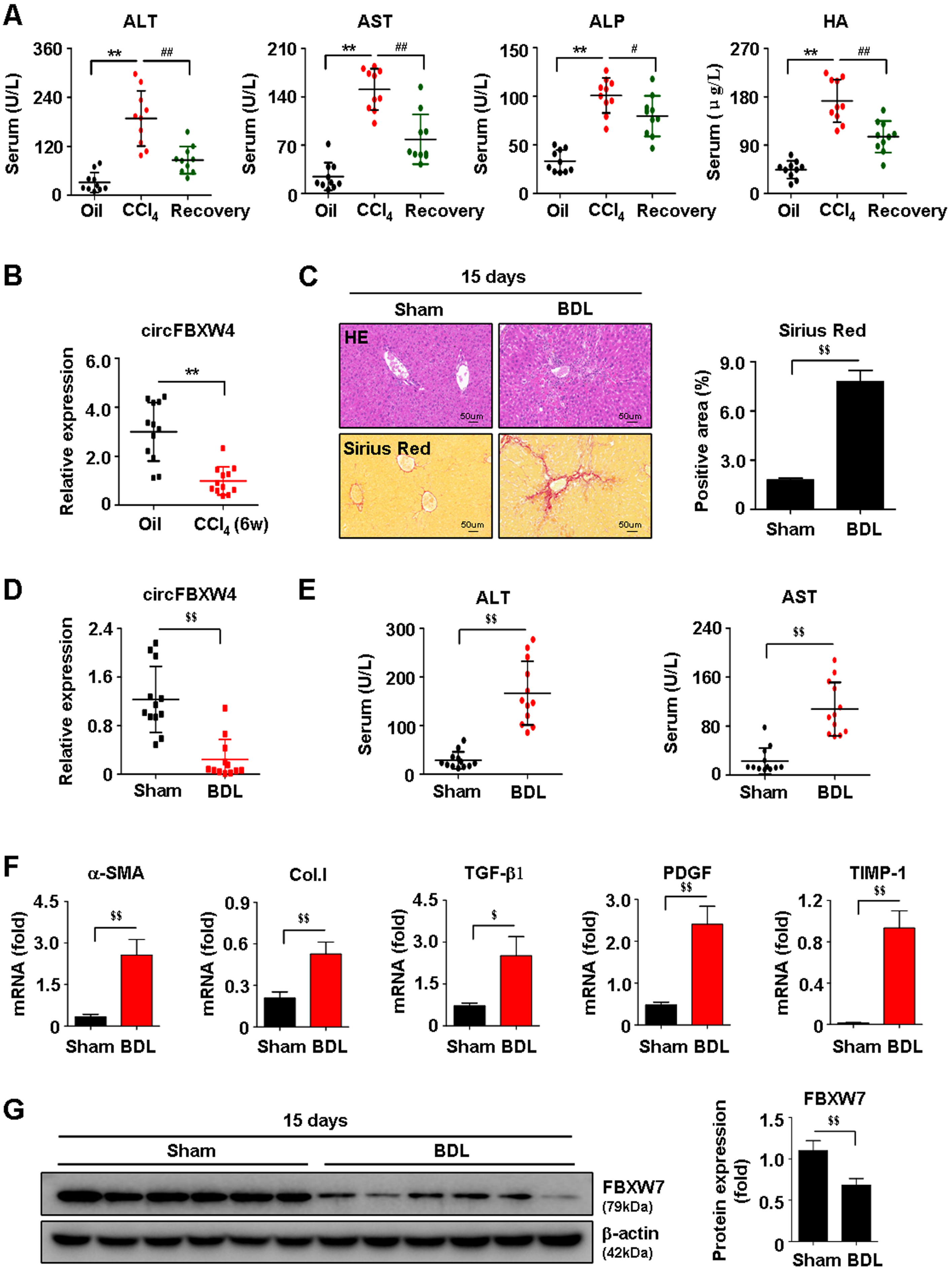

A
ALT
AST
ALP
HA
Serum (U/L)
Serum (μ g/L)
Oil
CCl4
Recovery
B
circFBXW4
Relative expression
Oil
CCl4 (6w)
C
15 days
Sham
BDL
HE
Sirius Red
50um
Positive area (%)
D
circFBXW4
E
ALT
AST
F
α-SMA
Col.I
TGF-β1
PDGF
TIMP-1
mRNA (fold)
G
15 days
FBXW7
(79kDa)
β-actin
(42kDa)
Protein expression (fold)

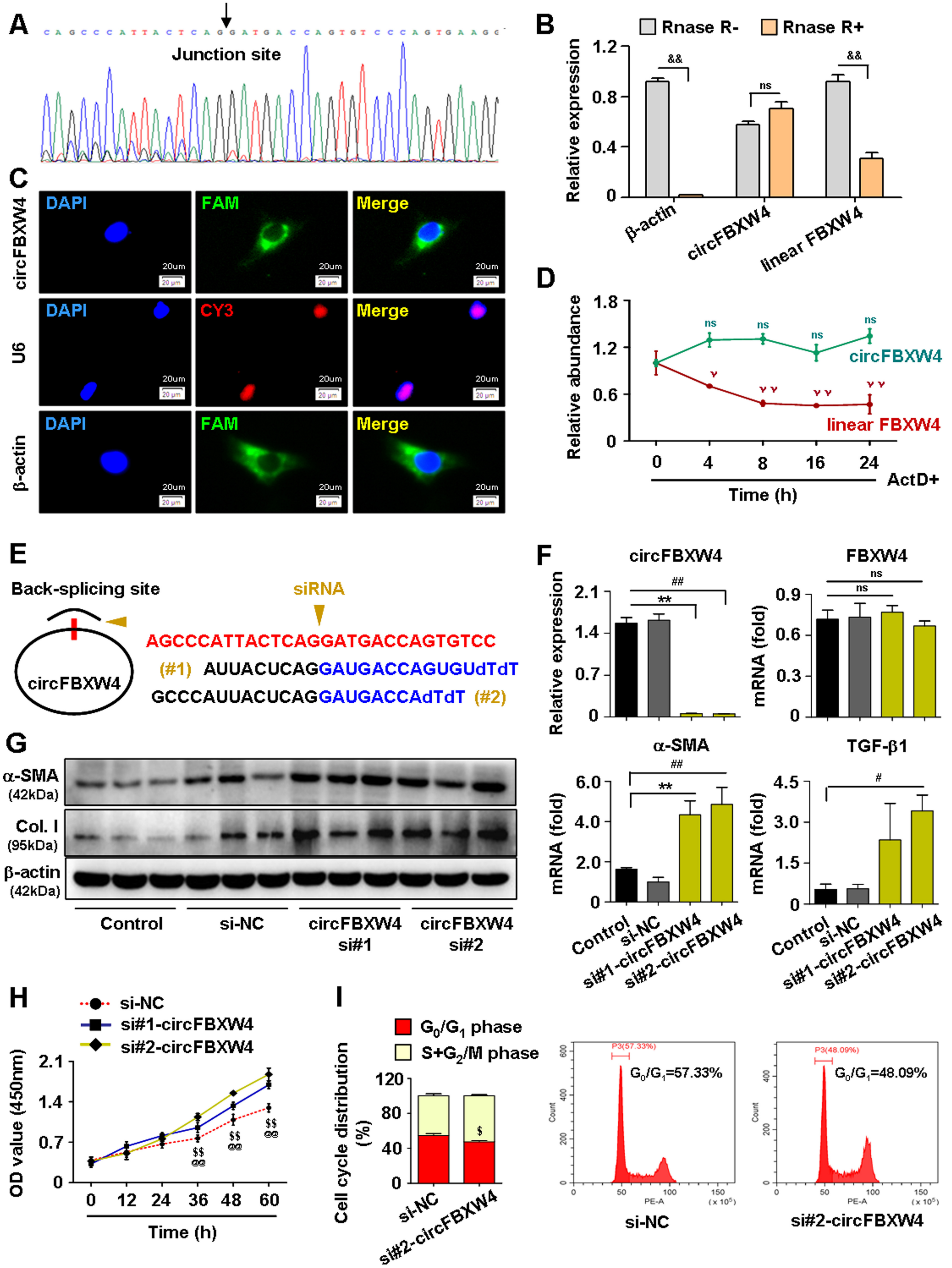
A
Junction site
B
Rnase R-
Rnase R+
Relative expression
β-actin
circFBXW4
linear FBXW4
C
circFBXW4
U6
β-actin
DAPI
FAM
CY3
Merge
20um
D
Relative abundance
circFBXW4
linear FBXW4
Time (h)
ActD+
E
Back-splicing site
siRNA
circFBXW4
AGCCCATTACTCAGGATGACCAGTGTCC
(#1) AUUACUCAGGAUGACCAGUGUdTdT
GCCCAUUACUCAGGAUGACCAdTdT (#2)
F
circFBXW4
FBXW4
α-SMA
TGF-β1
Relative expression
mRNA (fold)
Control
si-NC
si#1-circFBXW4
si#2-circFBXW4
G
α-SMA (42kDa)
Col. I (95kDa)
β-actin (42kDa)
Control
si-NC
circFBXW4 si#1
circFBXW4 si#2
H
si-NC
si#1-circFBXW4
si#2-circFBXW4
OD value (450nm)
Time (h)
I
G0/G1 phase
S+G2/M phase
Cell cycle distribution (%)
si-NC
si#2-circFBXW4
P3(57.33%)
G0/G1=57.33%
P3(48.09%)
G0/G1=48.09%
Count
PE-A

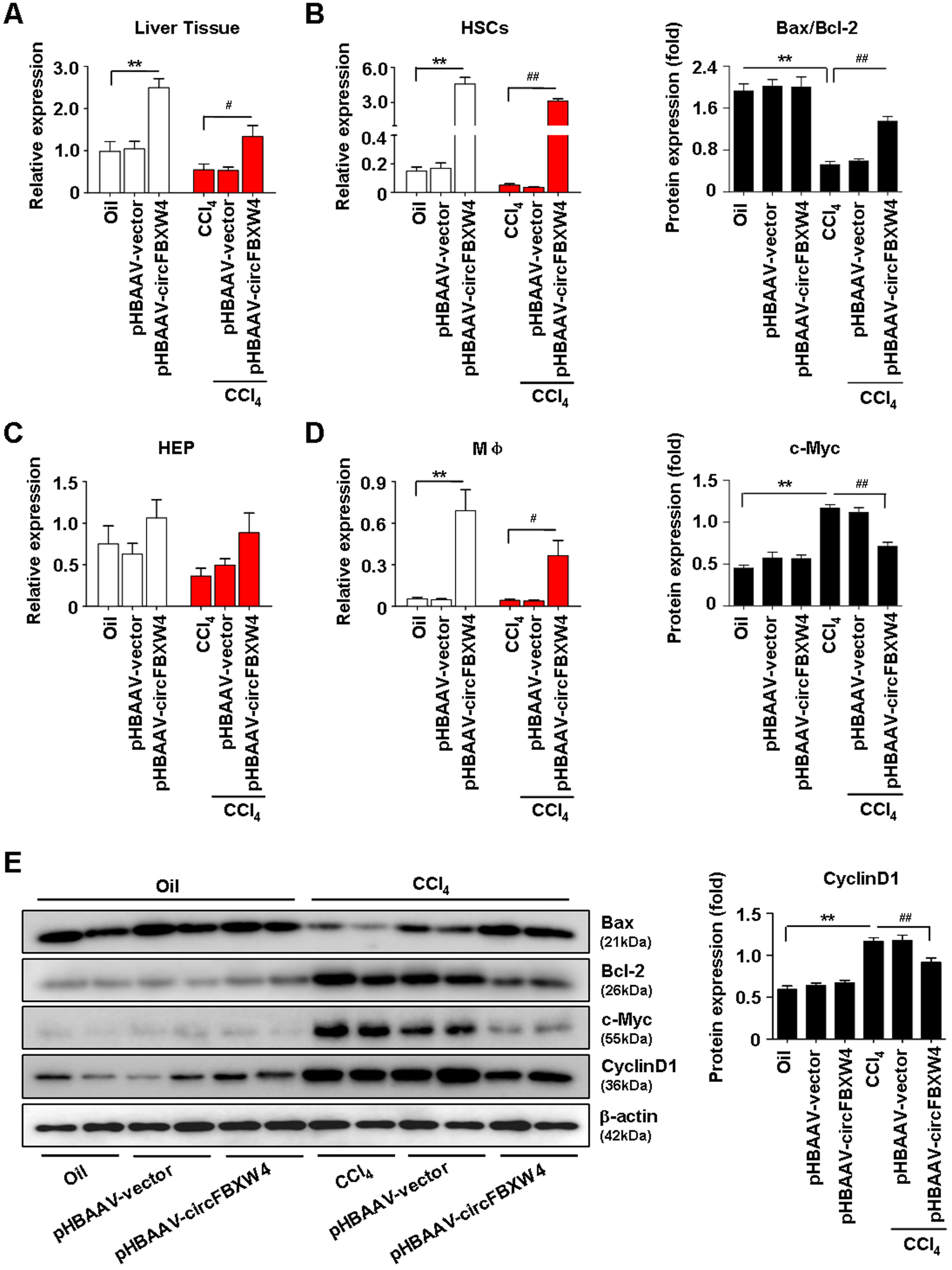

A
Liver Tissue
Relative expression
Oil
pHBAAV-vector
pHBAAV-circFBXW4
CCl4
B
HSCs
Bax/Bcl-2
Protein expression (fold)
C
HEP
D
M Φ
c-Myc
E
Bax (21kDa)
Bcl-2 (26kDa)
c-Myc (55kDa)
CyclinD1 (36kDa)
β-actin (42kDa)
CyclinD1

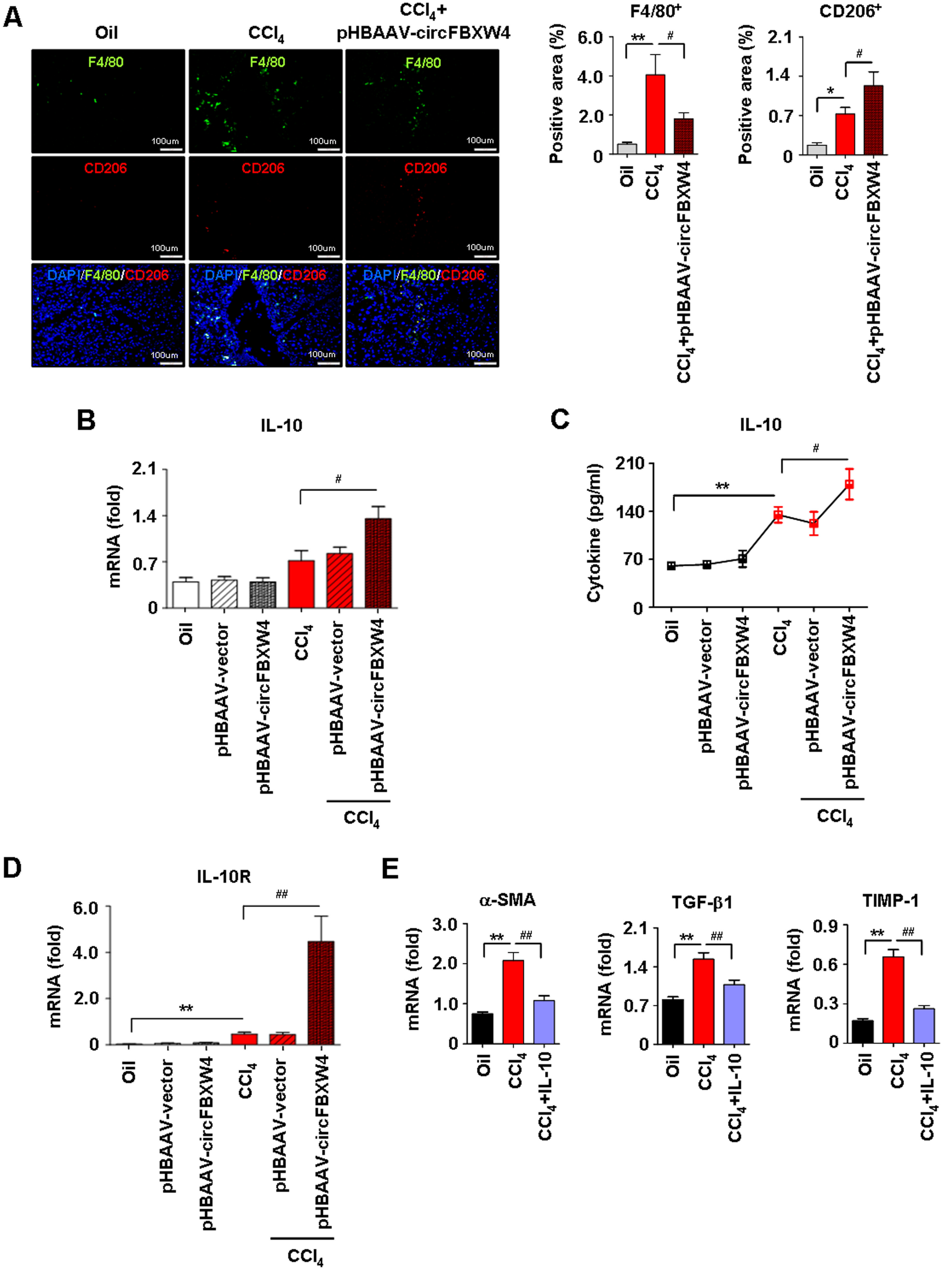

A
Oil
CCl4
CCl4+ pHBAAV-circFBXW4
F4/80
CD206
DAPI/F4/80/CD206
100um
F4/80+
CD206+
Positive area (%)
Oil
CCl4
CCl4+pHBAAV-circFBXW4
B
IL-10
mRNA (fold)
Oil
pHBAAV-vector
pHBAAV-circFBXW4
CCl4
C
IL-10
Cytokine (pg/ml)
D
IL-10R
mRNA (fold)
E
α-SMA
TGF-β1
TIMP-1
CCl4+IL-10

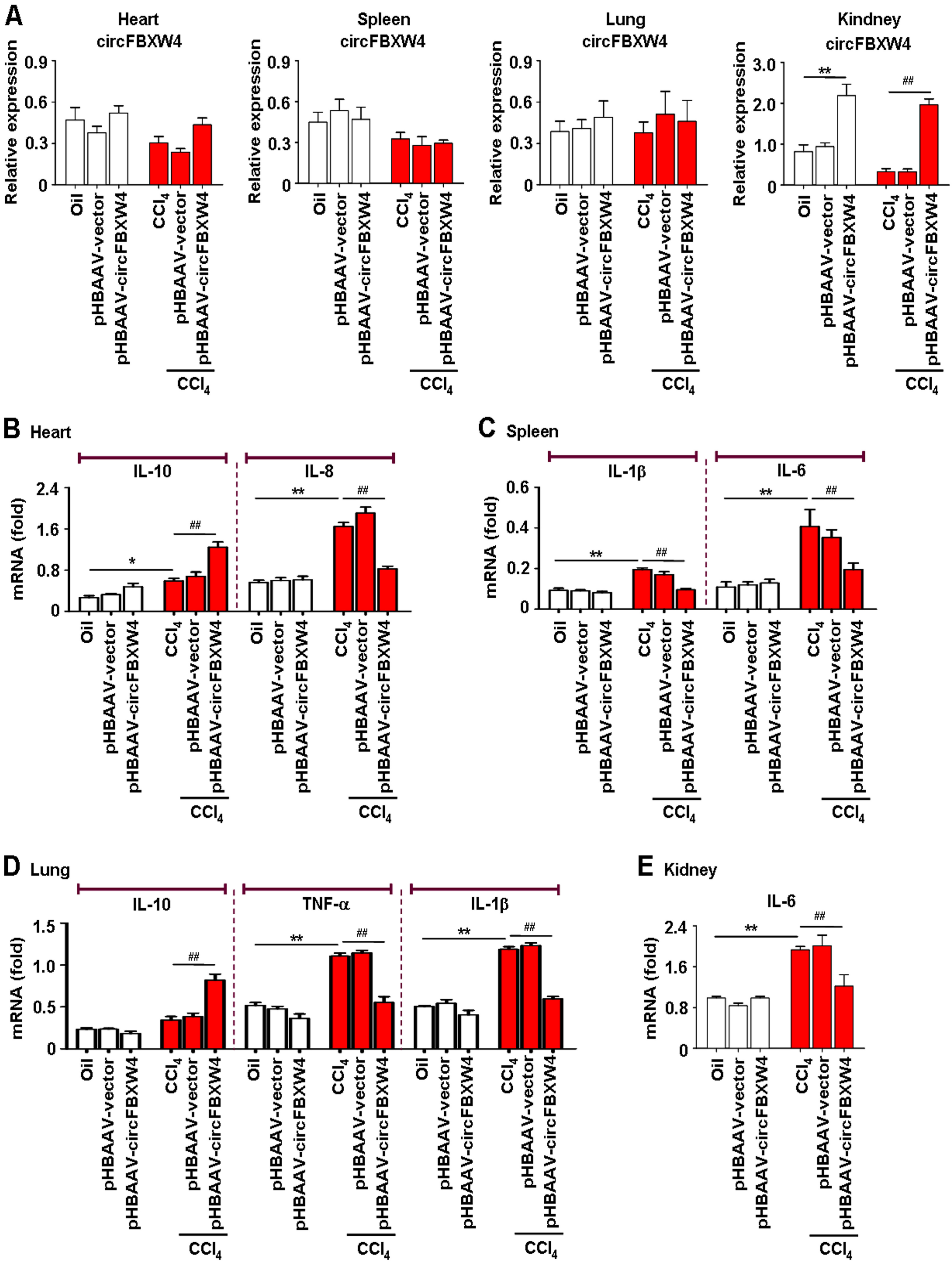

A
Heart circFBXW4
Spleen circFBXW4
Lung circFBXW4
Kidney circFBXW4
Relative expression
Oil
pHBAAV-vector
pHBAAV-circFBXW4
CCl4
B Heart
IL-10
IL-8
mRNA (fold)
C Spleen
IL-1β
IL-6
D Lung
IL-10
TNF-α
IL-1β
E Kidney
IL-6

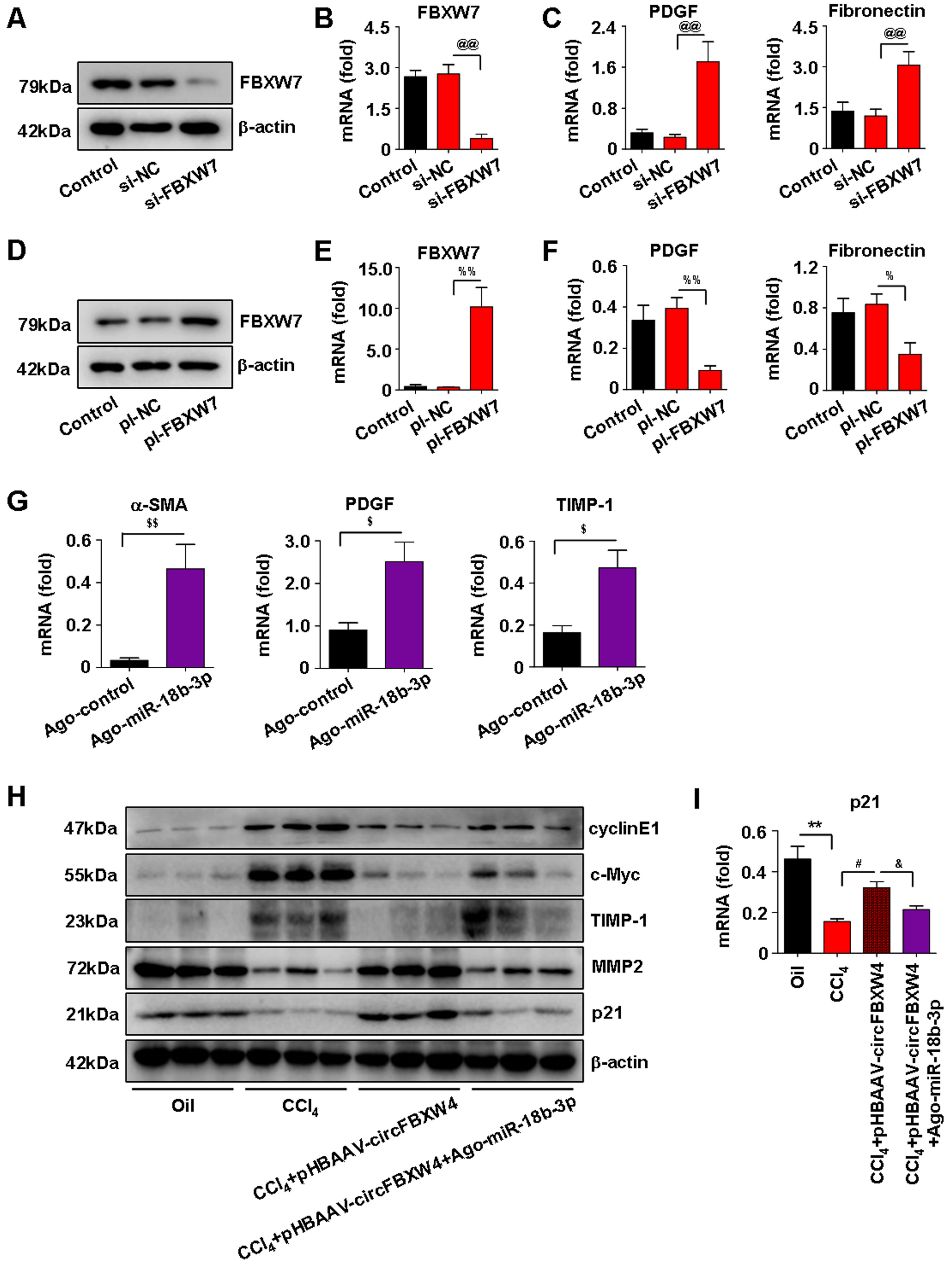

A
79kDa
42kDa
FBXW7
β-actin
Control
si-NC
si-FBXW7
B
FBXW7
mRNA (fold)
@@
C
PDGF
Fibronectin
@@
D
pl-NC
pl-FBXW7
E
%%
F
%
G
α-SMA
PDGF
TIMP-1
$$
$
Ago-control
Ago-miR-18b-3p
H
47kDa
55kDa
23kDa
72kDa
21kDa
42kDa
cyclinE1
c-Myc
TIMP-1
MMP2
p21
β-actin
Oil
CCl4
CCl4+pHBAAV-circFBXW4
CCl4+pHBAAV-circFBXW4+Ago-miR-18b-3p
I
p21
**
#
&

Fig. 1C

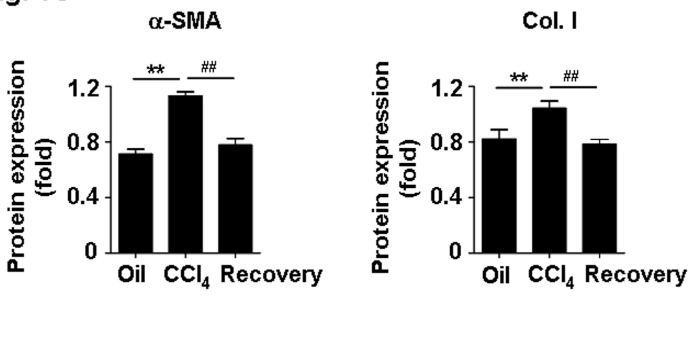

Fig. 4B

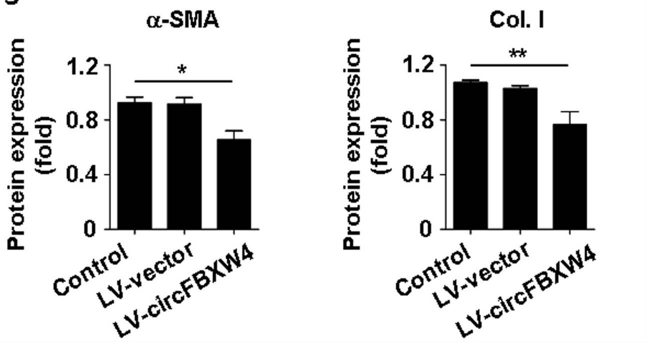

Fig. 5D

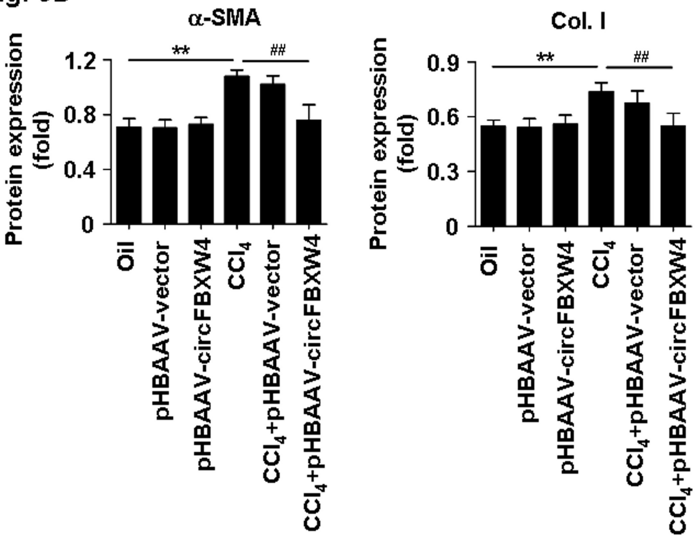

Fig. 8H

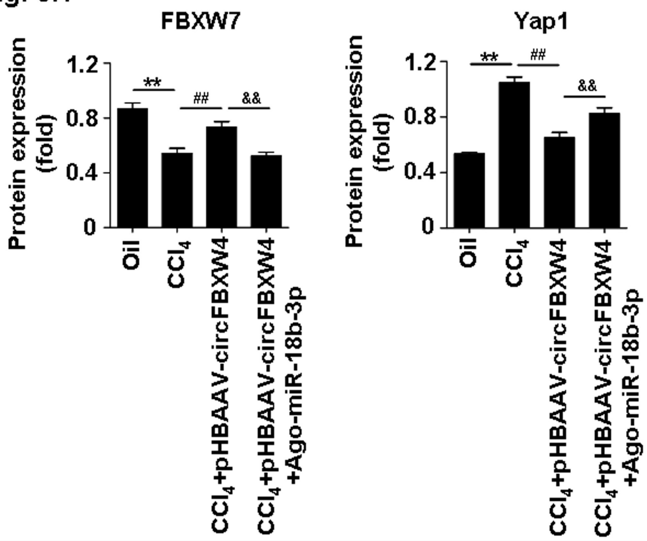

Supplementary Fig. 2G

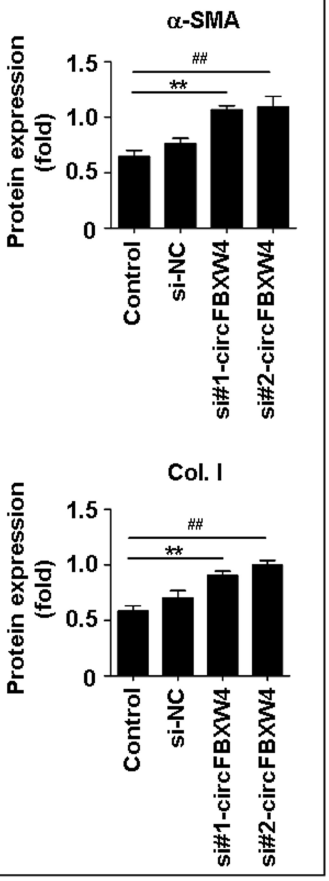

Supplementary Fig. 6D

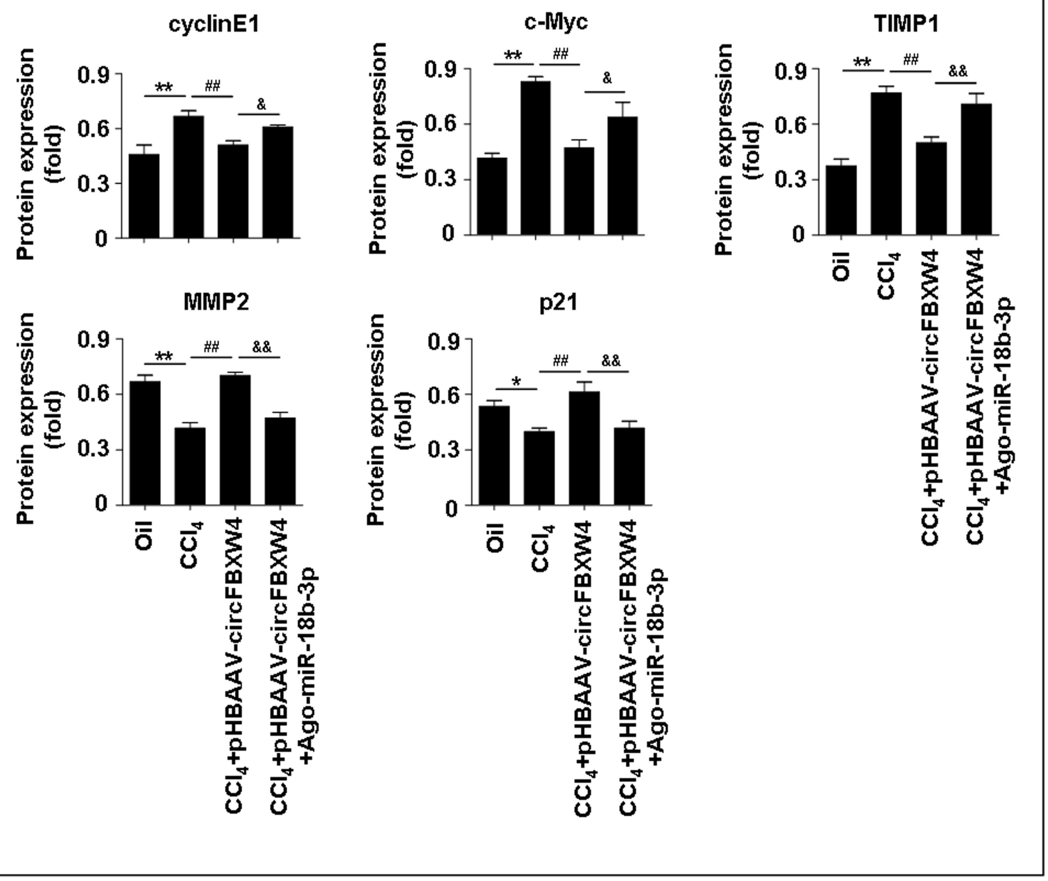

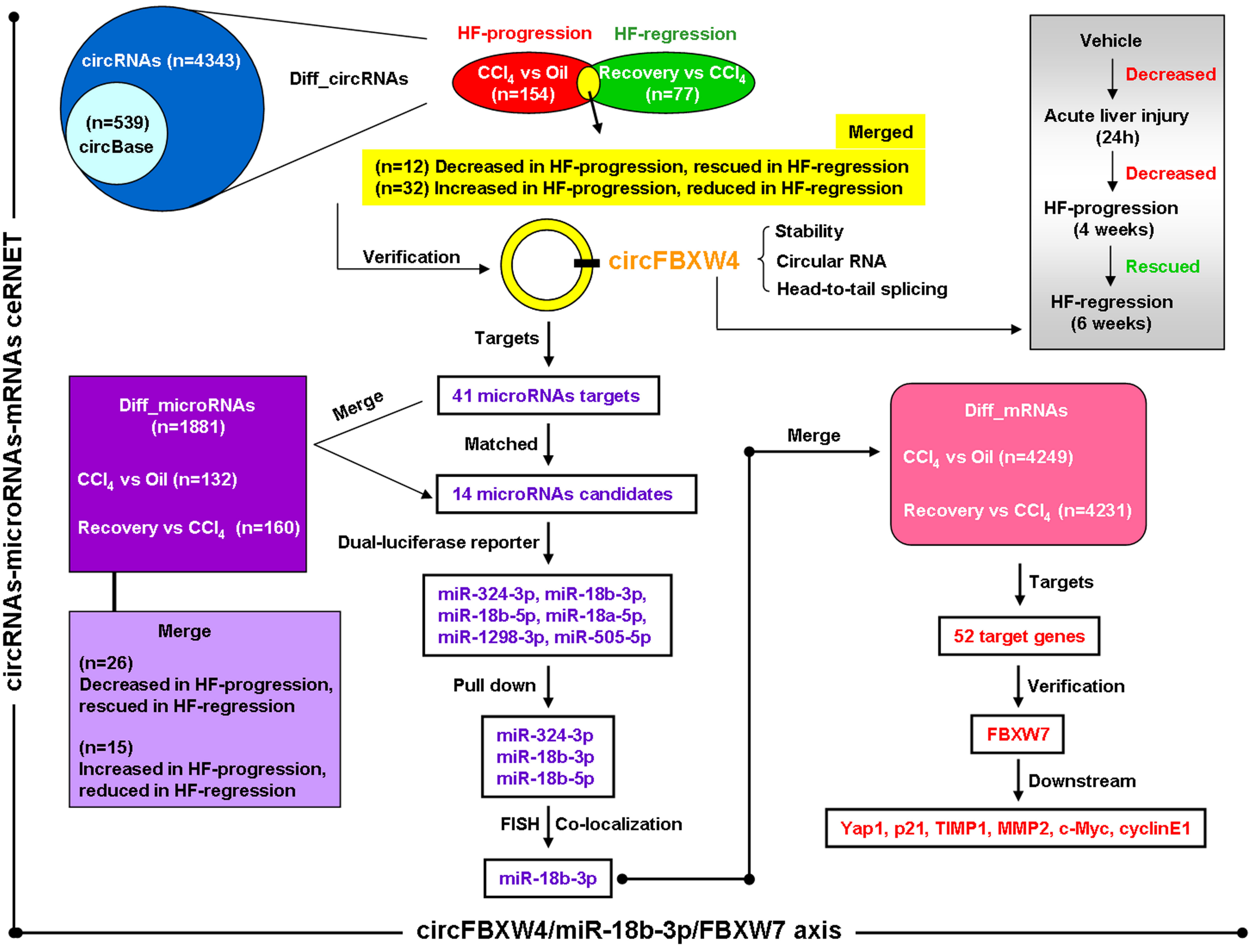

circRNAs-microRNAs-mRNAs ceRNET
circRNAs (n=4343)
(n=539) circBase
Diff_circRNAs
HF-progression
HF-regression
CCl_4 vs Oil (n=154)
Recovery vs CCl_4 (n=77)
Merged
(n=12) Decreased in HF-progression, rescued in HF-regression
(n=32) Increased in HF-progression, reduced in HF-regression
Verification
circFBXW4
Stability
Circular RNA
Head-to-tail splicing
Vehicle
Decreased
Acute liver injury (24h)
Decreased
HF-progression (4 weeks)
Rescued
HF-regression (6 weeks)
Targets
41 microRNAs targets
Matched
14 microRNAs candidates
Dual-luciferase reporter
miR-324-3p, miR-18b-3p, miR-18b-5p, miR-18a-5p, miR-1298-3p, miR-505-5p
Pull down
miR-324-3p
miR-18b-3p
miR-18b-5p
FISH
Co-localization
miR-18b-3p
Diff_microRNAs (n=1881)
CCl_4 vs Oil (n=132)
Recovery vs CCl_4 (n=160)
Merge
Merge
(n=26)
Decreased in HF-progression, rescued in HF-regression
(n=15)
Increased in HF-progression, reduced in HF-regression
Merge
Diff_mRNAs
CCl_4 vs Oil (n=4249)
Recovery vs CCl_4 (n=4231)
Targets
52 target genes
Verification
FBXW7
Downstream
Yap1, p21, TIMP1, MMP2, c-Myc, cyclinE1
circFBXW4/miR-18b-3p/FBXW7 axis

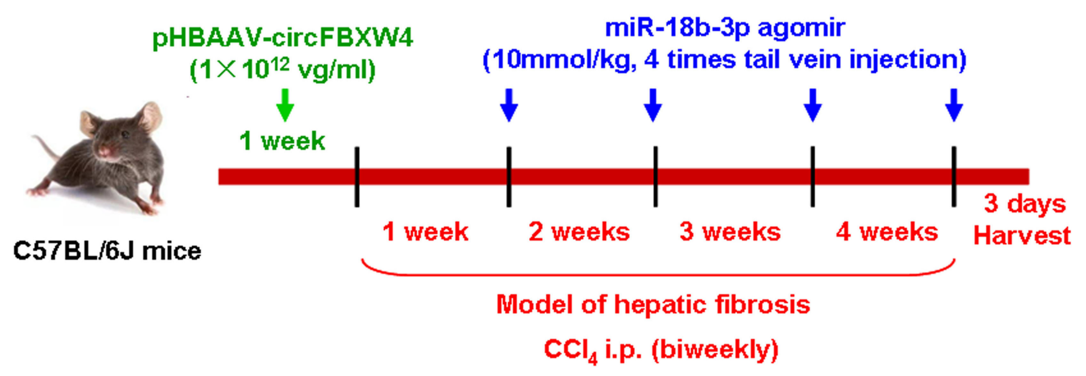
pHBAAV-circFBXW4
(1×10¹² vg/ml)
1 week
miR-18b-3p agomir
(10mmol/kg, 4 times tail vein injection)
C57BL/6J mice
1 week
2 weeks
3 weeks
4 weeks
3 days
Harvest
Model of hepatic fibrosis
CCl4 i.p. (biweekly)

Supplementary Table S1. Dysregulated circRNAs in liver fibrogensis.

circRNAs	Locus	Type	Host Gene	Regulation
mm9_circ_001729	Chr9	Exon	Rasa2	Down
mm9_circ_006286	Chr3	Exon	Elf2	Down
mm9_circ_014796	Chr5	Exon	Ep400	Down
mm9_circ_010464	Chr6	Exon	Ezh2	Down
mm9_circ_017500	Chr8	Exon	Irf2	Down
mm9_circ_005864	Chr13	Exon	Cdk13	Down
mm9_circ_003709	Chr14	Exon	Kat6b	Down
mm9_circ_018395	Chr16	Exon	Acap2	Down
mm9_circ_012897	Chr18	Exon	Atp9b	Down
mm9_circ_000338	Chr19	Exon	Fbxw4	Down
mm9_circ_005659	Chr1	Exon	Eprs	Down
mm9_circ_016411	Chr2	Exon	Rprd1b	Down
mm9_circ_014393	Chr5	Exon	Zfp644	Down
mm9_circ_004990	Chr5	Exon	Srpk2	Down
mm9_circ_018254	Chr9	Exon	Armc8	Down
mm9_circ_009396	Chr6	Exon	Mkln1	Down
mm9_circ_002457	Chr5	Exon	Ube2k	Up
mm9_circ_013054	Chr1	Exon	Hecw2	Up
mm9_circ_005799	Chr11	Exon	Fxr2	Up
mm9_circ_003298	Chr5	Exon	Rbm33	Up
mm9_circ_004870	Chr9	Exon	Rasa2	Up
mm9_circ_016460	Chr17	Exon	Sos1	Up
mm9_circ_007735	Chr19	Exon	Fam160b1	Up
mm9_circ_008117	Chr7	Exon	Mettl9	Up
mm9_circ_017115	Chr11	Exon	Nmt1	Up
mm9_circ_007736	Chr4	Exon	Teme245	Up

mm9_circ_006238	Chr8	Exon	Psd3	Up

Supplementary Table S2. Primers, RNA and probes sequences used in this study.

1. Primers for qRT-PCR (mouse)

Terms	Forward primer (5'-3')	Reverse primer (5'-3')
mm9_circ_002457	GGAGCACCAGTTTCTAGTCCA	CTGGAGGTCCTGCTATTTCTCC
mm9_circ_013054	TGCCAGAAACCACTCACTCA	CTGCCACGTTGTGGTTCTGT
mm9_circ_005799	CAGGACTGTCATGGACGGAG	GAACCCTCACCACACCAGAC
mm9_circ_004870	GGTTTTCACTGCAGCCGATT	TTTCAACAACCTGGGTGCGA
mm9_circ_016460	TGTGTACCACTGTCTGCATT	TAGACTCAAGAGTAGGGTGAACT
mm9_circ_007735	TTGAAACCGTGGAGGCAGTT	TGACCTTTTGGGCCATCCTC
mm9_circ_008117	ATCCTGGCATTGGTTTTGCC	CACAAACATGGAGCCTCTTCC
mm9_circ_017115	CCGATTCGACTATTCCCCAGA	GTCCCTGACCCACTGAGAAC
mm9_circ_007736	GGTGAACAATACGGCTGTGA	GGGTGATTTGCTAGAGTTTCGT
mm9_circ_003298	GTGGGACCCCAAAGATTTCC	CGTCCCTGTTTATGCTGTCGT
mm9_circ_006238	CAAGGAGTAAATGAAGGTGGGGA	GTTTTCCCCTCAGCAAAGCA
mm9_circ_001729	ACAGAGAATGGAACCGTGTG	ACAATCGCGCATTTTGTTGG
mm9_circ_006286	GAGCCAGAAGGAAGACAATGC	TCACTGTTTTCACAACTCCCAA
mm9_circ_014796	CAGCTTCCGCCAAATGTTTCT	TGGTGCATTATCACCTCCCTT
mm9_circ_010464	GAGAGTGTGACCCTGACCTC	AACAAACCGGTCCCTTCTCA
mm9_circ_017500	TGAGTATGCGGTCCTGACTT	CGCATTCGCATCCGTTCC
mm9_circ_005864	GCAGATTTTGGACTTGCTCGTT	GGCTTCTGCTTCGGGATTTG
mm9_circ_003709	GATCATGCCCCAGCACCAA	AGTCACGAGCTCCTTGTTTCC
mm9_circ_018395	ATCTGTTCAAACGAGCCAGCA	GGGCCTTAATTGATCTCTGGGT
mm9_circ_012897	TCTGGTAGTGTCCTGCTCACA	TGGCATTTCATCCAAATGGGC
mm9_circ_000338	CATCCAGACCGAAGACCGAG	CCGCCAGTTCTGAGACAGTT
mm9_circ_016411	GGTGGAATACACCCAGAACCA	CTCAGGTCCTTTCCTTTTACTGTT
mm9_circ_004990	AATCCAACTATCAAGGCCTCCC	AAGGAGCTTTCTGTTGAGGCT
mm9_circ_014393	TCCGTTGATCTATCAGCCACA	GCACCAGTAATGTCGGTGTTT
mm9_circ_005659	AGTGCTGGGAAAAGATGAGGA	ACGAACTGTAGGAAACCGTGG

mm9_circ_018254	CCTGCATCGTGGTTCCAGTA	AACCGCACCTTGACACTAGA
mm9_circ_009396	TGCTTGCGAGGAGTTGATTGA	CTTGGGGAGGATAGTTGCTCT
FBXW4	CCGGACAGAAAGAGATGCAGT	ATAGCAGGACTGAGGAACAGC
FBXW7	TAGCCAAGGTCCAAGAAGTAGC	CACCCACAAGTTCACCATTC
α-SMA	CGGGCTTTGCTGGTGATG	CCCTCGATGGATGGGAAA
Collagens I	GCTCCTCTTAGGGGCCACT	CCACGTCTCACCATTGGGG
TIMP-1	GCAACTCGGACCTGGTCATAA	CGGCCCGTGATGAGAAACT
PDGF	CATCCGCTCCTTTGATGATCTT	GTGCTCGGGTCATGTTCAAGT
TGF-β1	CTCCCGTGGCTTCTAGTGC	GCCTTAGTTTGGACAGGATCTG
Yap1	GAGGGACTCCGAATGCAG	CGAGAGTGATAGGTGCCACTG
p21	CAAAGTGTGCCGTTGTCTCTT	TCAAAGTTCCACCGTTCTCG
TNF-α	CACCACCATCAAGGACTCAA	AGGCAACCTGACCACTCTCC
IL-1β	CTTTGAAGTTGACGGACCC	TGAGTGATACTGCCTGCCTG
IL-6	GAGGATACCACTCCCAACAGACC	AAGTGCATCATCGTTGTTCATACA
IL-8	GGTCTTCCTGCTTGAATGGCTTGA	GCGGTGTCCTGATTATCGTCCTC
IL-10	GCCTTGCAGAAAAGAGAGCT	AAAGAAAGTCTTCACCTGGC
IL-10R	AGGCAGAGGCAGCAGGCCCAGCAGAATGCT	TGGAGCCTGGCTAGCTGGTCACAGTAGGTCT
CCL2	GGGCCTGCTGTTCACAGTT	CCAGCCTACTCATTGGGAT
CCL9	CCCTCTCCTTCCTCATTCTTACA	AGTCTTGAAAGCCCATGTGAAA
GAPDH	GGACCTCATGGCCTACATGG	TAGGGCCTCTCTTGCTCAGT

2. Primers for qRT-PCR (human)

Terms	Forward primer (5'-3')	Reverse primer (5'-3')
hsa_circ_0008362	ACTCACAATGATGCCCCCTT	CGAGAGGGGATTCTGCTGAA
FBXW4	GCATCTCTATGCTGCCCTGT	AAGCCTCTAGTGCAAGGTGC
α-SMA	GTGTTGCCCCTGAAGAGCAT	GCTGGGACATTGAAAGTCTCA
Collagens I	CCCGGGTTTCAGAGACAACTTC	TCCACATGCTTTATTCCAGCAATC
TIMP-1	CTTCTGCAATTCCGACCTCGT	ACGCTGGTATAAGGTGGTCTG
TGF-β1	GGCCAGATCCTGTCCAAGC	GTGGGTTTCCACCATTAGCAC
p21	TGCCCAAGCTCTACCTTCC	CAGGTCCACATGGTCTTCCT

β-actin	GCCAACACAGTGCTGTCTGG	CTCAGGAGGAGCAATGATCTTG

3. Primers for PCR

circFBXW4	Sense (5'-3')	Antisense (5'-3')
Divergent primers	TGTGGACTGTAAAGGTGGCATCA	TGGTAGGCCAGGATGAAATTAGC
Convergent primers	ATCGGATTCTGCTGAAGTGGAG	GAAGGTGCTGTGGATCTTGTGAA

4. siRNA sequences used for circFBXW4 knockdown.

siRNA	Forward primer (5'-3')	Reverse primer (5'-3')
si-circFBXW4#1	GCCCAUUACUCAGGAUGACCAdTdT	UGGUCAUCCUGAGUAAUGGGCdTdT
si-circFBXW4#2	AUUACUCAGGAUGACCAGUGUdTdT	ACACUGGUCAUCCUGAGUAAUdTdT
Negative control (NC)	UUCUCCGAACGUGUCACGUdTdT	ACGUGACACGUUCGGAGAAdTdT

5. siRNA sequences used for FBXW7 knockdown.

siRNA	Forward primer (5'-3')	Reverse primer (5'-3')
si-FBXW7	CCAUGUUCAGCAACACCAAdTdT	UUGGUGUUGCUGAACAUGGdTdT
Negative control (NC)	UUCUCCGAACGUGUCACGUdTdT	ACGUGACACGUUCGGAGAAdTdT

6. Probes sequences used for FISH.

Probes	(5'-3')
circFBXW4 (FAM)	GCTCCTTTACTGGAATGCCTGCCATGCTGAGTAATGGGCTG
miR-18b-3p (CY3)	AGAAGGGGCATTTAGGGCAGTA

7. Probes sequences used for pull-down.

Probes	(5'-3')
circFBXW4	GCTCCTTTACTGGAATGCCTGCCATGCTGAGTAATGGGCTG

NC	UUGUACUACACAAAAGUACUG

8. RNA oligo sequences.

Agomir	Forward primer (5'-3')	Reverse primer (5'-3')
miR-18b-3p agomir	UACUGCCCUAAAUGCCCCUUCU	AAGGGGCAUUUAGGGCAGUAUU
NC agomir	UUCUCCGAACGUGUCACGUTT	ACGUGACACGUUCGGAGAATT

9. Sequences for luciferase reporter.

Group	Terms	Sequences
-	CircFBXW4-wt	CATGGCAGGCATTCCAGTAAAGGAGCGCGTGAAACTGTCTCAGAACTGGC<br>GGCTGGGGCGCTGCCGAGATCGGATTCTGCTGAAGTGGAGATATAGTCAG<br>ATGCCTTGGATGCAGCTACAGGATGCTTCTCTGTACCTATCACAGGCTAATT<br>TCATCCTGGCCTACCAGTTCCGCCCGGATGGTGCAAGCTTGAACCGACGG<br>CCCTTCAGAGTCTTCTCTGGGCATGATGAGGACGTCTGTCACTTTGTGCTG<br>GCCAACTCCCACATCGTCAGTGCAGGAGGAGACGGGAAGATCGGTGTTCA<br>CAAGATCCACAGCACCTTCACTGTCAAGTACTCGGCTCACGAGCAGGAGG<br>TGAACTGTGTGGACTGTAAAGGTGGCATCATTGTGAGTGGCTCCCGGGAC<br>AGGACAGCCAAGGTATGGCCTCTGGCCTCGGGCCGGCTGGGGCAGTGCTT<br>ACACACCATCCAGACCGAAGACCGAGTCTGGTCCATTGCTATCAGCCCATT<br>ACTCAG
	miR-NC mimics sense	UUCUCCGAACGUGUCACGUdTdT
	miR-NC mimics antisense	ACGUGACACGUUCGGAGAAdTdT
1	CircFBXW4-mut	CATGGCAGGCATTCCAGTAAAGGAGCGCGTGAAACTGTCTCAGAACTGGC<br>GGCTGGGGCGCTGCCGAGATCGGATTCTGCTGAAGTGGAGATATAGTCAG<br>ATGCCTTGGATGCAGCTACAGGATGCTTCTCTGTACCTATCACAGGCTAATT<br>TCATCCTGGCCTACCAGTTCCGCCCGGATGGTGCAAGCTTGAACCGACGG<br>CCCTTCAGAGTCTTCTCTGGGCATGATGAGGACGaCaGTgAgTTTGaGaTGGa<br>CAACTCCCACATCGTCAGTGCAGGAGGAGACGGGAAGATCGGTGTTCACA<br>AGATCCACAGCACCTTCACTGTCAAGTACTCGGCTCACGAGCAGGAGGTG<br>AACTGTGTGGACTGTAAAGGTGGCATCATTGTGAGTGGCTCCCGGGACAG<br>GACAGCCAAGGTATGGCCTCTGGCCTCGGGCCGGCTGGGGCAGTGCTTAC<br>ACACCATCCAGACCGAAGACCGAGTCTGGTCCATTGCTATCAGCCCATTAC<br>TCAG
	miR-18a-5p mimics sense	UAAGGUGCAUCUAGUGCAGAUAG
	miR-18a-5p mimics antisense	AUCUGCACUAGAUGCACCUUAUU
2	CircFBXW4-mut	CATGGCAGGCATTCCAGTAAAGGAGCGCGTGAAACTGTCTCAGAACTGGC<br>GGCTGGGGCGCTGCCGAGATCGGATTCTGCTGAAGTGGAGATATAGTCAG

		ATGCCTTGGATGCAGCTACAGGATGCTTCTCTGTACCTATCACAGGCTAATT<br>TCATCCTGGCCTACCAGTTCCGCCCGGATGGTGCAAGCTTGAACCGACGG<br>CCCTTCAGAGTCTTCTCTGGGCATGATGAGGACGTCTGTCACTTTGTGCTG<br>GCCAACTCCCACATCGTCAGTGCAGGAGGAGACGGGAAGATCGGTGTTCA<br>CAAGATCCACAGCACCTTCACTGTCAAGTACTCGGCTCACGAGCAGGAGG<br>TGAACTGTGTGGACTGTAAAGGTGGCATCATTGTGAGTGGCTCCCGGGAC<br>AGGACAGCCAAGGTATGGCCTCTGGCCTCGGGCCGGCTGtGtCtGaGCTTAC<br>ACACCATCCAGACCGAAGACCGAGTCTGGTCCATTGCTATCAGCCCATTAC<br>TCAG
	miR-18b-3p mimics sense	UACUGCCCUAAAUGCCCCUUCU
	miR-18b-3p mimics antisense	AAGGGGCAUUUAGGGCAGUAUU
3	CircFBXW4-mut	CATGGCAGGCATTCCAGTAAAGGAGCGCGTGAAACTGTCTCAGAACTGGC<br>GGCTGGGGCGCTGCCGAGATCGGATTCTGCTGAAGTGGAGATATAGTCAG<br>ATGCCTTGGATGaAtCTtCAGtAaGaTTCTCTGTcCaTATCACAGGCTAATTTCA<br>TCCTGGCCTACCAGTTCCGCCCGGATGGTGCAAGCTTGAACCGACGGCCC<br>TTCAGAGTCTTCTCTGGGCATGATGAGGACGTCTGTCACTTTGTGCTGGCC<br>AACTCCCACATCGTCAGTGCAGGAGGAGACGGGAAGATCGGTGTTCACAA<br>GATCCACAGCACCTTCACTGTCAAGTACTCGGCTCACGAGCAGGAGGTGA<br>ACTGTGTGGACTGTAAAGGTGGCATCATTGTGAGTGGCTCCCGGGACAGG<br>ACAGCCAAGGTATGGCCTCTGGCCTCGGGCCGGCTGGGGCAGTGCTTACA<br>CACCATCCAGACCGAAGACCGAGTCTGGTCCATTGCTATCAGCCCATTACT<br>CAG
	miR-18b-5p mimics sense	UAAGGUGCAUCUAGUGCUGUUAG
	miR-18b-5p mimics antisense	AACAGCACUAGAUGCACCUUAUU
4	CircFBXW4-mut	CATGGCAGGCATTCCAGTAAAGGAGCGCGTGAAACTGTCTCAGAACTGGC<br>GGCTGGGGCGCTGCCGAGATCGGATTCTGCTGAAGTGGAGATATAGTCAG<br>ATGCCTTGGATGCAGCTACAGGATGCTTCTCTGTACCTATCACAGGCTAATT<br>TCATCCTGGCCTACCAGTTCCGCCCGGATGGTGCAAGCTTGAACCGACGG<br>CCCTTCAGAGTCTTCTCTGGGCATGATGAGGACGTCTGTCACTTTGTGCTG<br>GCCAACTCCCACATCGTCAGTGCAGGAGGAGACGGGAAGATCGGTGTTCA<br>CAAGATCCACAGCACCTTCACTGTCAAGTACTCGGCTCACGAGCAGGAGG<br>TGAACTGTGTGGACTGTAAAGGTGGCATCATTGTGAGTGGCTCCCGGGAC<br>AGGACAGCCAAGGTATGGCCTCTGtCCTCGGtCaGGaTcGtGgAcTcCTTACAC<br>ACCATCCAGACCGAAGACCGAGTCTGGTCCATTGCTATCAGCCCATTACTC<br>AG
	miR-324-3p mimics sense	CCACUGCCCCAGGUGCUGCU
	miR-324-3p mimics antisense	CAGCACCUGGGGCAGUGGUU
5	CircFBXW4-mut	CATGGCAGGCATTCCAGTAAAGGAGCGCGTGAAACTGTCTCAGAACTGGC<br>GGCTGGGGCGCTGCCGAGATCGGATTCTGCTGAAGTGGAGATATAGTCAG<br>ATGCCTTGGATGCAGCTACAGGATGCTTCTCTGTACCTATCACAGGCTAATT<br>TCATCCTGGCCTACCAGTTCCGCCCGGATGGTGCAAGCTTGAACCGACGG<br>CCCTTCAGAGTCTTCTCTGGGCATGATGAGGACGTCTGTCACTTTGTGCTG<br>GCCAACTCCCACATCGTCAGTGCAGGAGGAGACGGGAAGATCGGTGTTCA

		CAAGATCCACAGCACCTTCACTGTCAAGTACTCGGCTCACGAGCAGGAGGTGAACTGTGTGGACTGTAAAGGTGGaAgCcTTGaGAGaGtCaCaCGGGACAGGACAGCCAAGGTATGGCCTCTGGCCTCGGGCCGGCTGGGGCAGTGCTTACACACCATCCAGACCGAAGACCGAGTCTGGTCCATTGCTATCAGCCCATTACTCAG
	miR-505-5p mimics sense	GGGAGCCAGGAAGUAUUGAUGUU
	miR-505-5p mimics antisense	CAUCAAUACUUCCUGGCUCCCUU
6	CircFBXW4-mut	CATGGCAGGCATTCCAGTAAAGGAGCGCGTGAAACTGTCTCAGAACTGGCGGCTGGGGCGCTGCCGAGATCGGATTCTGCTGAAGTGGAGATATAGTCAGATGCCTTGGATGCAGCTACAGGATGCTTCTCTGTACCTATCACAGGCTAATTTCATCCTGGCCTACgAcTaCCtCaCGtAaGGTGCAAGCTTGAACCGACGGCCCTTCAGAGTCTTCTCTGGGCATGATGAGGACGTCTGTCACTTTGTGCTGGCCAACTCCCACATCGTCAGTGCAGGAGGAGACGGGAAGATCGGTGTTCACAAGATCCACAGCACCTTCACTGTCAAGTACTCGGCTCACGAGCAGGAGGTGAACTGTGTGGACTGTAAAGGTGGCATCATTGTGAGTGGCTCCCGGGACAGGACAGCCAAGGTATGGCCTCTGGCCTCGGGCCGGCTGGGGCAGTGCTTACACACCATCCAGACCGAAGACCGAGTCTGGTCCATTGCTATCAGCCCATTACTCAG
	miR-1298-3p mimics sense	CAUCUGGGCAACUGAUUGAACU
	miR-1298-3p mimics antisense	UUCAAUCAGUUGCCCAGAUGUU

Supplementary Table S3. Characteristics of the subjects enrolled in this study.

Parameter	Patients	
Number, *n*	14	
Gender, *n* (%)	Male	8 (57.1%)
	Female	6 (42.9%)
Age (yr)*	47.57 ± 9.25	
BMI > 25, *n* (%)	3 (21.4%)	
ALT (U/L)*	76.79 ± 40.35	
AST (U/L)*	46.07 ± 26.67	
Albmumin (g/L)*	35.45 ± 4.59	
Prothrombin time (%)*	89.21 ± 15.71	
Platelet count ($\times 10^9$/L)*	164.29 ± 29.72	
Hyaluronic acid (μg/L)*	185.35 ± 66.35	

Abbreviations: BMI, body mass index; ALT, alanine aminotransferase; AST, aspartate transaminase. *Mean ± SD.
